# Function-structure approach reveals novel insights on the interplay of Immunoglobulin G 1 proteoforms and Fc gamma receptor IIa allotypes

**DOI:** 10.3389/fimmu.2023.1260446

**Published:** 2023-09-18

**Authors:** Steffen Lippold, Karishma Mistry, Sunidhi Lenka, Kevin Whang, Peilu Liu, Sebastian Pitschi, Felix Kuhne, Dietmar Reusch, Lance Cadang, Alexander Knaupp, Saeed Izadi, Alexis Dunkle, Feng Yang, Tilman Schlothauer

**Affiliations:** ^1^ Protein Analytical Chemistry, Genentech, A Member of the Roche Group, South San Francisco, CA, United States; ^2^ Biological Technologies, Genentech, A Member of the Roche Group, South San Francisco, CA, United States; ^3^ Pharmaceutical Development, Genentech, A Member of The Roche Group, South San Francisco, CA, United States; ^4^ Pharma Technical Development Europe, Roche Diagnostics GmbH, Penzberg, Germany; ^5^ Pharma Research and Early Development, Roche Innovation Center Munich, Penzberg, Germany

**Keywords:** affinity chromatograghy, CD32 (FcgRII), ADCP, deamidation, molecular dynamics, mass spectrometry, glycosylation, critical quality attribute (CQA)

## Abstract

Human Fc gamma receptor IIa (FcγRIIa) or CD32a has two major allotypes with a single amino acid difference at position 131 (histidine or arginine). Differences in FcγRIIa allotypes are known to impact immunological responses such as the clinical outcome of therapeutic monoclonal antibodies (mAbs). FcγRIIa is involved in antibody-dependent cellular phagocytosis (ADCP), which is an important contributor to the mechanism-of-action of mAbs by driving phagocytic clearance of cancer cells. Hence, understanding the impact of individual mAb proteoforms on the binding to FcγRIIa, and its different allotypes, is crucial for defining meaningful critical quality attributes (CQAs). Here, we report a function-structure based approach guided by novel FcγRIIa affinity chromatography-mass spectrometry (AC-MS) assays to assess individual IgG1 proteoforms. This allowed to unravel allotype-specific differences of IgG1 proteoforms on FcγRIIa binding. FcγRIIa AC-MS confirmed and refined structure-function relationships of IgG1 glycoform interactions. For example, the positive impact of afucosylation was higher than galactosylation for FcγRIIa Arg compared to FcγRIIa His. Moreover, we observed FcγRIIa allotype-opposing and IgG1 proteoform integrity-dependent differences in the binding response of stress-induced IgG1 proteoforms comprising asparagine 325 deamidation. The FcγRIIa-allotype dependent binding differences resolved by AC-MS were in line with functional ADCP-surrogate bioassay models. The molecular basis of the observed allotype specificity and proteoform selectivity upon asparagine 325 deamidation was elucidated using molecular dynamics. The observed differences were attributed to the contributions of an inter-molecular salt bridge between IgG1 and FcγRIIa Arg and the contribution of an intra-molecular hydrophobic pocket in IgG1. Our work highlights the unprecedented structural and functional resolution of AC-MS approaches along with predictive biological significance of observed affinity differences within relevant cell-based methods. This makes FcγRIIa AC-MS an invaluable tool to streamline the CQA assessment of therapeutic mAbs.

## Introduction

Fc gamma receptors (FcγRs) mediate key immunological responses by interacting with the fragment crystallizable (Fc) part of Immunoglobulins (Ig) (1). FcγRIIa (CD32a) is the most widespread activating FcγR and is present on most leukocytes (monocytes, neutrophils, eosinophils, basophils, mast cells) and platelets (2, 3). Only primates express FcγRIIa and human FcγRIIa shows two major polymorphisms at position 131 (histidine or arginine) (4). The FcγRIIa His/Arg polymorphisms (His/His, His/Arg, Arg/Arg) show ethnic-dependent variants and were linked to differences in disease susceptibilities and efficacy of therapeutic antibodies (2, 5–8). The functional relevance of the FcγRIIa polymorphism was attributed to distinct affinity differences of FcγRIIa to IgG subclasses. FcγRIIa contributes to macrophage-mediated antibody-dependent cellular cytotoxicity (ADCC) and antibody-dependent cellular phagocytosis (ADCP) (9). The clinical relevance of FcγRIIa makes the understanding of IgG – FcγRIIa interactions crucial for defining critical quality attributes (CQAs) of therapeutic antibodies to ensure their safety and efficacy.

Both IgG and FcγRIIa are complex glycoproteins. IgG1 comprises a conserved *N*-glycosylation site at Asn 297 and FcγRIIa is glycosylated at Asn 61 and Asn 142. The binding affinity of the IgG1-FcγRIIa interaction is around 1 µM (10). The lower hinge and CH2 domain of IgG1 interact asymmetrically with FcγRIIa (11). FcγRIIa glycosylation does not directly contribute to the IgG1 interaction, and a recent study found no impact of the FcγRIIa glycan macro- or micro-heterogeneity on IgG1 glycoform selectivity (11, 12). In contrast, IgG1 Asn 297 glycosylation features are known to moderately affect the affinity to FcγRIIa and ADCP (13–15). Numerous studies on the FcγRIIa affinity rankings of IgG1 glycosylation features were performed, but the findings are often contradictory (9, 13, 16–28). Of note, deglycosylation of natural IgG1 leads to drastically decreased FcγR binding, including FcγRIIa (16, 17). Besides Fc glycosylation, understanding the impact of other post-translational modifications (PTMs), such as deamidation, on FcγRIIa binding and function is needed to define CQAs of therapeutic antibodies, but is currently understudied and inconsistent (17, 29–31). Stress studies are commonly performed to evaluate potential impacts on the safety and efficacy profile of therapeutic mAbs using relevant stress conditions, representative for storage or manufacturing, and forced stress conditions to induce high levels of PTMs (17). In particular, thermal stress is highly relevant and may induce high level of PTMs, clipping variants and aggregates. Interestingly, thermal stress has been shown to selectively enrich deamidation at Asn 325 (VSNK motif) under mildly acidic conditions in IgG1 (32, 33). Asn 325 is a known hotspot for deamidation, which decreases the efficacy of mAbs due to reduced FcγRIIIa binding and ADCC (33–37).

The functional *in-vivo* response of FcγR – IgG interactions is mediated by avidity through immune complexes upon target binding (1, 38). However, the highly increased complexity of *in-vivo* experiments and immune complexes lead to highly impaired sensitivity and precision for an assessment of individual IgG-FcγR interactions (39). *In-vitro* studies of monomeric IgG-FcγR interaction have been widely accepted for *in-vivo* predictions. Most *in-vitro* IgG-FcγR affinity assessments apply surface plasmon resonance spectroscopy (SPR). However, SPR studies may show discrepancies due to the complex interaction and the inherent molecular heterogeneity of the IgG and FcγR (10). The sensitivity, precision and accuracy of SPR is largely affected by the presence of co-existing IgG proteoforms, i.e., the combination of post-translational modifications (PTMs) at the intact protein level, and in particular aggregates (17). In contrast, affinity chromatography (AC) allows the relative binding assessment of complex proteoform mixtures. Proteoforms comprising functionally relevant PTMs are chromatographically separated based on the interaction to an immobilized, functionally relevant interaction partner, e.g., FcγRIIIa for predicting ADCC activity (18). Hence, AC is gaining momentum as a tool for studying IgG-FcR interactions (18, 37, 40, 41). Moreover, AC coupled to mass spectrometry (MS) techniques provides unprecedented insights into structure-function relationships (42). MS may be applied using offline analysis of peptides obtained from enzymatic digestion of AC fractions (bottom-up approach), which provides the highest structural resolution and site-specificity, but the information on the initial proteoform heterogeneity is compromised. In addition, online AC-MS hyphenation offers a direct intact mass readout and is highly useful for PTMs with distinct mass differences such as glycoforms. So far, AC-MS methods for studying IgG-FcR interactions have been developed for FcRn (half-life) and FcγRIII (ADCC) and relied on bottom-up analysis of affinity fractions or online intact mass analysis (40, 41, 43). The expansion of the AC-MS toolbox is of high importance to redefine the structural and functional understanding of FcR-IgG interactions in a proteoform-resolved manner.

This study reports on the development of two novel AC-MS assays, covering both FcγRIIa allotypes, as key technology for expanding the ADCP function-structure understanding of IgG1 proteoforms. For this, we established MS-compatible chromatographic conditions allowing to separate and identify IgG1 proteoforms that were functionally different with respect to ADCP-mediated potency. In addition to refining the IgG1 glycoform affinity ranking, we demonstrated FcγRIIa allotype-specific differences of IgG1 proteoforms induced by thermal stress. Our new findings from FcγRIIa AC-MS were substantiated by orthogonal binding assays and cell line-based bioassays, and the underlying structural mechanisms were elucidated by molecular dynamics.

## Materials and methods

### Antibodies

All antibodies used in this study were produced in-house for research and development purposes. In total, five different mAbs were used named mAb1 to mAb5. Two glycoengineered versions of mAb1 with high levels of afucosylation or galactosylation were used. Afucosylated mAb1 was produced in an ɑ1,6 fucosyltransferase knockout CHO cell line, while galactosylated mAb1 was generated by incubating purified mAb1 samples with bovine β1,4 galactosyltransferase in the presence of MnCl_2_ and UDP galactose. Six glycoengineered (G1F/G1F, G1/G1, G2F/G2F, G2/G2, G2FS2/G2FS2, G2S2/G2S2) versions of mAb2 were prepared using TransGLYCIT (Genovis, Sweden) following the manufacturer instructions. Two additional versions of mAb2 containing high levels of α-2,3- or 2,6-linked sialic acids were obtained by *in-vitro* glycoengineering as described elsewhere (44). In addition, an Fc mutant of mAb4 comprising Asn 325 Asp was used. All antibodies were produced in CHO cells. Thermal stress conditions were applied for mAb1 mAb4 for up to 8 weeks at 40°C in the corresponding formulation buffer at pH 5.5 (e.g., for mAb1: 20 mM L-histidine acetate, 240 mM sucrose, 10 mM L-methionine, 0.04% (w/v) polysorbate 20, pH 5.5).

### FcγRIIa affinity column preparation

The FcγRIIa columns were produced in-house. An Fc (PGLALA mutant) fusion construct with the extra-cellular domain of FcγRIIa (His or Arg) and C-terminal Avi-tag was transiently expressed in HEK 293-F cells (Thermo Fisher) (**Figure S1**). The selected fusion protein presents FcγRIIa as dimer, which is expected to be more relevant for structure-function interpretations (11). The construct was *in-vivo* biotinylated by BirA ligase co-expression and immobilized by incubation with streptavidin agarose beads (Cytiva) overnight. The receptor density of the beads was set to 1 mg/mL. The functionalized sepharose was packed into a tricorn column housing (5 mm x 20 mm, Cytiva).

### FcγRIIa affinity chromatography mass spectrometry

FcγRIIa affinity chromatography-mass spectrometry was performed on a Vanquish Horizon (Thermo Scientific) connected to a Q Exactive UHMR Orbitrap (Thermo Scientific). The mobile phases consisted of 150 mM ammonium acetate (mobile phase A) and 150 mM acetic acid (mobile phase B). The column temperature was set to 25°C and the flow rate was kept at 0.25 mL/min. The UV signal was acquired at 280 nm. Samples were prepared for analysis by buffer-exchanging to mobile phase A (10 kDa molecular weight cutoff filter, Merck). Upon injection (10 µg, 1 mg/mL), a 5 min isocratic step using 100% mobile phase A was applied. Then, a linear gradient of 15 min to 80% mobile phase B was used for elution. Next, the column was washed with 80% mobile phase B for 5 min before returning to the starting condition. Between runs, 15 min of re-equilibration using 100% mobile phase A was used. Flow-splitting (approx. 2 µL/min to MS) was applied for electrospray ionization using a Flex ion source (Thermo scientific). MS data acquisition was performed in positive ion mode (2 kV capillary voltage). The *m/z* range was set from 2,000 Th to 15,000 Th and resolution to 25,000. For improved declustering, desolvation voltage (-175V) and in-source collision induced dissociation energy (30V) were applied. For each data point, 10 micro scans were averaged, resulting in a scan rate of 1.6 scans/sec. Charge deconvolution of intact mass spectra was performed using UniDec (version 5.2.1) (45). Assignments of IgG glycoforms was performed manually upon deconvolution and were based on the statistically most likely glycoforms. Of note, additional isomeric glycoform pairings (e.g., G1F/G1F vs. G0F/G2F) were not listed as additional options. Extracted ion chromatograms (EICs) of specific proteoforms were generated using the theoretical *m/z* values of charge states 21+ to 25+ with a tolerance of 100 ppm in Freestyle (v.1.8, Thermo Scientific).

### Bottom-up sample preparation

Antibody samples (100 µg each) were denatured by addition of 6M Guanidine HCl, 360 mM Tris, 2 mM EDTA, pH 7.0. Reduction was accomplished using 20 mM DTT and 30 min incubation at 37°C. Then, each sample was alkylated with 50 mM iodoacetamide protected from light at room temperature for 15 min. The alkylation reaction was quenched by adding additional 10 mM DTT. The reduced and alkylated samples were buffer-exchanged using Bio spin P-6 gel columns (Biorad) into digestion buffer (50 mM Tris, 2 mM CaCl2, pH 7.5). Lyophilized trypsin (sequencing grade, Promega) was reconstituted to 0.2 mg/mL with water. Digestion was initiated with the addition of 0.2 mg/mL trypsin solution to desalted antibody samples at a 1:20 enzyme-protein (w/w) ratio. The digestion was incubated at 37°C for 60 min in a water bath. Finally, digested samples were quenched with trifluoroacetic acid. For glycoproteomic analysis of FcγRIIa, 100 µg of each construct, FcγRIIa His and FcγRIIa Arg, were buffer exchanged (10 kDa Amicon Filter, Merck) to 25 mM Tris, 1mM CaCL_2_ at pH 8.8 and a concentration of 1 µg/µL. The analytes were reduced using 5 mM DTT for 30 min at 60°C. Next, cysteine aminoethylation was performed by adding 10 mM 2-bromoethylamine (Sigma Aldrich) and incubation for 60 min at 60°C. Next, 5 mM DTT was added to the solution to quench the alkylation. Finally, 1 µg (1:100 w/w) of trypsin (sequencing grade, Promega) was added to each sample and the digestion was performed overnight (16 h – 18 h) at 37°C.

### Bottom-up liquid chromatography-MS/MS analysis

Tryptic digested samples were separated using a 150 mm × 2.1 mm, 1.7 μm Waters Acquity BEH C18 column with UV detection at 214 nm. Mobile phase A was 0.1% trifluoroacetic acid or formic acid in water (v/v) and mobile phase B was 0.08% trifluoroacetic acid or formic acid in acetonitrile (v/v). The LC gradient started at 100% mobile phase A. Mobile phase B was elevated to 40% from 14 to 47 min, and then column was washed with 95% mobile phase B for 4 min before returning to initial condition (mAbs). Mobile phase B was kept at 1% for the first two minutes and then linearly increased to 13% at 7 min, followed by a linear gradient to 35% mobile phase B at 42 min and a 2 min wash step using 95% mobile phase B. Column temperature for LC-MS/MS analysis of mAbs was maintained at 60°C with a flow rate of 0.3 mL/min and 77°C with 0.2 mL/min flow rate for FcγRIIa. The injection volume of the protein digest was 20 µL (mAbs) and 10 µL (FcγRIIa). LC-MS/MS experiments were performed using a Thermo Fisher Scientific Q Exactive Plus mass spectrometer (mAbs) or Thermo Fisher Scientific Orbitrap Fusion Lumos (FcγRIIa) operated in positive ion mode. The spay voltage was 3.5 kV, the ion transfer tube temperature was 320°C/250°C, and the sheath and auxiliary gas flow rate were 30 and 6/10, respectively (mAbs/FcγRIIa). For mAb analysis, full-scan MS1 spectra were acquired using a mass range of 200 – 2000 Th with a resolution of 35,000, 100 ms maximum ion injection time and an AGC target of 4E5. Data-dependent fragmentation with top 8 ions was induced by higher-energy collisional dissociation (HCD) fragmentation using normalized collision energy (NCE) of 27%. An isolation window of 2.5 Th was applied, the maximum injection time was set to 50 ms and an AGC of 1E5 was used. The MS2 resolution was set to 17,500. For FcγRIIa (glyco-)peptides, full-scan MS1 mass spectra were collected using a mass range of 120 – 3,500 Th, a resolution of 120,000, 100 ms ion injection time and an AGC target of 4E5. MS2 data of FcγRIIa (glyco-)peptides were acquired by data-dependent HCD (NCE = 28%) in an *m/z* range of 100 – 2,000. Charge states 2 – 8 were included for fragmentation. An isolation window of 3 Th, AGC target of 1E5, maximum injection time of 50 ms and a resolution of 30,000 was used. In addition, stepped-HCD (combined spectrum of NCE = 20%, 30% and 50%) was triggered by oxonium ion presence (204.0867, 366.1396).

### Post-translational modifications data analysis

Peptides were identified based on accurate mass and MS/MS using Protein Metrics software. Relative quantitation was performed manually by XCalibur software. Extracted ion chromatograms (EICs) were obtained for each expected peptide using the monoisotopic value from the most abundant charge state. The relative percentage of a chemically modified peptide was estimated by dividing the peak area of the modified peptide peak by the sum of the peak areas for the modified and unmodified peptide peaks.

The glycoproteomic data analysis of FcγRIIa peptides was performed as described previously (41). In short, one peptide moiety for each glycosylation site (**Figures S2**-**S5**) was selected for further evaluation based on MS/MS identification in Byonic (v.4.4 Protein Metrics). Then, an MS1-based search using expected retention time and mass differences was performed using GlycopeptideGraphMS (v.2.05) (46). A combined glycan list of all glycosylation sites was generated and each potential glycopeptide was manually checked of mass accuracy (< 10 ppm), retention time, isotopic pattern quality (idotp > 0.85) and integrated in Skyline (47). The relative abundances of glycans and non-glycopeptides were calculated for individual glycosylation sites based on total area normalization. Of note, the nomenclature of mAb glycans followed established mAb glycan nomenclature, whereas glycan compositions were used to describe FcγRIIa glycosylation (**Tables S1**, **S2**).

### Surface plasmon resonance spectroscopy

SPR experiments were performed on a Biacore T200 instrument (Cytiva). His-tagged FcγRIIa receptors were prepared in-house and captured on separate Biacore CM5 sensor chips with immobilized anti-His antibodies (Cytiva). The relative binding properties, at a defined part at the end of the association phase, of mAb1 and stressed mAb1 samples towards FcγRIIa His and FcγRIIa Arg were analyzed. Upon each sample injection and binding measurement, the chip surface was regenerated to remove all captured antibodies and analytes. The signals from a reference flow cell and from blank buffer injections were subtracted from the analyte signal, and data were evaluated using Biacore T200 Evaluation Software. As system suitability criteria, the CV (≤ 10%) and relative difference (≤ 10%) of control samples were used to ensure precision and accuracy. Representative SPR sensorgrams are provided in **Figure S6**.

### Cell-based potency and functional assays

Cell-based functional assays were performed using monocyte- and lymphocyte-based reporter cell lines able to detect the cross-linking and activation of FcγRIIa (CD32a) in response to mAb clustering. The monocyte-based assay was used for the functional assessment of enriched FcγRIIa His AC fractions with varying degrees of IgG1 Asn 325 deamidation levels to correlate retention time in AC with functionality. The monocyte activation assay utilized an in-house THP1 reporter cell line, a human monocyte cell line engineered to overexpress FcγRIIa His along with the luciferase enzyme downstream of an NF-κB response element. The lymphocyte-based assay was used to assess the biological relevance of FcγRIIa allotype-differences with respect to thermal stress of IgG1. The Fc effector reporter bioassay (Promega) utilized Jurkat reporter cells expressing NFAT-induced luciferase activity and either human FcγRIIa His or FcγRIIa Arg. The reporter cell lines allow the detection of FcγRIIa-dependent cellular activation by measuring luminescence, which is proportional to the amount of luciferase expressed and the activity of the reporter gene.

In all versions of the method, the mAb sample is coated at a high density on an assay plate via anti-Fab capture allowing for subsequent interaction of mAb1 Fc region with FcγRIIa and activation of the reporter cell. Briefly, the anti-Fab capture reagent (CaptureSelect™ Human Fab-kappa Kinetics Biotin Conjugate, Thermo Fisher Scientific) was coated on a high-binding 96 well assay plate at 10 µg/mL. The plate was washed (Phosphate-buffered saline, 0.05% Polysorbate 20) and blocked using assay medium (RPMI 1640, 10% HI FBS, 1x Glutamax, 1x Pen-Strep) for 1-2 h. A dilution series of mAb1 standard and samples (50 µL per well) was added to the plate for 30 min at 37°C to capture mAb1. Plates were washed of excess mAb1, and the reporter cells (50 µL per well) were added to the assay plate at a concentration of 1.25e6 cells/mL. After incubation at 37°C for 180-250 min, the luminescence substrate reagent (OneGlo, Promega), which lyses the cells, was added to the plate (50 µL per well) and luminescence (RLU) was recorded using a luminescence plate reader (Molecular Devices) after 15 min. Results were analyzed by comparing a mAb1 reference standard to the mAb1 sample using either a 4P logistic curve comparison to calculate relative potency or by calculating fold response.

### Molecular dynamics

The complex structure of FcγRIIa and human IgG1 Fc was obtained from the RCSB PDB database PDB ID: 3RY6 (11). Glycans were added to the Fc and FcγRIIa as reported in the crystal structure. A total of 6 systems were studied at pH 7: Fc WT with FcγRIIa Arg, Fc WT with FcγRIIa His, Fc double deamidation (Asn 325 Asp) with FcγRIIa Arg, Fc double deamidation (Asn 325 Asp) with FcγRIIa His, Fc single deamidation (Asn 325 Asp) with FcγRIIa Arg, Fc single deamidation (Asn 325 Asp) with FcγRIIa His. The protein was build using ff19SB (48) forcefield and Glycam (49) force field parameters were used for glycans. The protein was solvated in a truncated water box of 10 Å from the protein and OPC water model was used (50). Counterions were added to neutralize the system. All the simulations were performed in Amber 20 (51). The initial structure was generated by the prepareforleap module of Amber and then tleap was used (52). Minimization was done on the starting solvated structure with 1000 steps of steepest descent and 4000 steps of conjugate gradient with positional restraint on the protein with a force constant of 10.0 kcal/(mol.Å^2^). Subsequently the system was heated with the temperature increased gradually from 10 K to 300 K for 0.3 ns at constant volume using langevin thermostat and a frictional coefficient of 5 ps^-1^. The system was then subjected to 1.3 ns NVT equilibration and then 8 ns of constant pressure and temperature equilibration using monte carlo barostat with the protein restrained by a force constant of 1.0 kcal/(mol.Å^2^). The final round of relaxation involved 10 ns of constant volume and temperature with no restraints. Five replicates of 300 ns production run were conducted using NVT ensemble. These five replicates have different random seeds and starting points to account for convergence. The hydrogen bond distances and contact analysis was done using cpptraj module of Amber and the protein images were created using VMD 1.9.2 (53). The plots were made using matplotlib of python. Histograms were obtained using a Gaussian kernel density estimator in python.

## Results and discussion

### Both recombinant FcγRIIa allotypes show comparable glycosylation profiles

**Figure 1:**
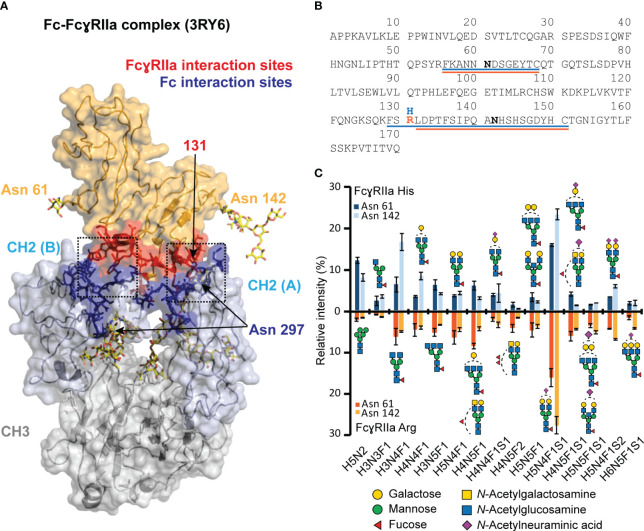
Visualization of FcγRIIa - Fc interaction and glycoproteomic profile of FcγRIIa His and Arg allotypes. **(A)** FcγRIIa-Fc interaction (3RY6) highlighting the glycosylation sites as well as interaction sites (11). **(B)** Sequence of extracellular domains of FcγRIIa allotypes highlighting tryptic glycopeptides for His (blue) and Arg (orange) allotype. **(C)** Glycoproteomic comparison of most abundant glycans found on FcγRIIa allotypes and glycosylation sites. Suitable tryptic glycopeptides (cleavage C-terminal to Lys, Arg, Cys) were obtained by applying cysteine aminoethylation as reported previously (54). A complete list of all identified and quantified glycan compositions is listed in **Table S1**. Glycan structure assignments are based on MS/MS information and Hayes et al. (55) Error bars represent standard deviation of technical replicates (n = 3).Of note, the residue numbering of His/Arg 131 was based on Sondermann et al. (56).

Currently, there is no evidence that FcγRIIa glycosylation (Asn 61, Asn 142) may impact the IgG1-FcγRIIa interaction or affinity ranking of Fc glycosylation features (12). This is expected as the FcγRIIa glycosylation sites are outside of the FcγRIIa - IgG1 interaction (**Figure 1A**). However, differences in FcγR glycosylation are generally known to be a contributing factor to the large variety of reported affinity differences for IgG FcγR interactions (10). Hence, it is best practice to report the receptor glycosylation for IgG FcγR binding assays. The site-specific glycosylation profiles of both FcγRIIa allotype materials used in this study are highly comparable, allowing to relate all observed differences in the binding behavior solely to the FcγRIIa allotype, i.e., the difference in a single amino acid (**Figures 1B, C**). In total, 49 (FcγRIIa His) and 53 (FcγRIIa Arg) glycan compositions were identified and quantified for Asn 61. Asn 142 revealed 42 (FcγRIIa His) and 58 (FcγRIIa Arg) glycan compositions (**Table S1**). The major glycan composition for both glycosylation sites was H5N4F1S1 (**Figure 1C**). Other highly abundant glycan compositions included H3N4F1, H5N4F1S2, H5N2 and H4N4F1. Our findings on HEK-derived FcγRIIa glycosylation showed a high level of sialylated di-antennary complex-type structures, in line with previous findings on FcγRIIa derived from either HEK cells or primary human monocytes (55, 57).

### FcγRIIa affinity chromatography mass – spectrometry assay

**Figure 2:**
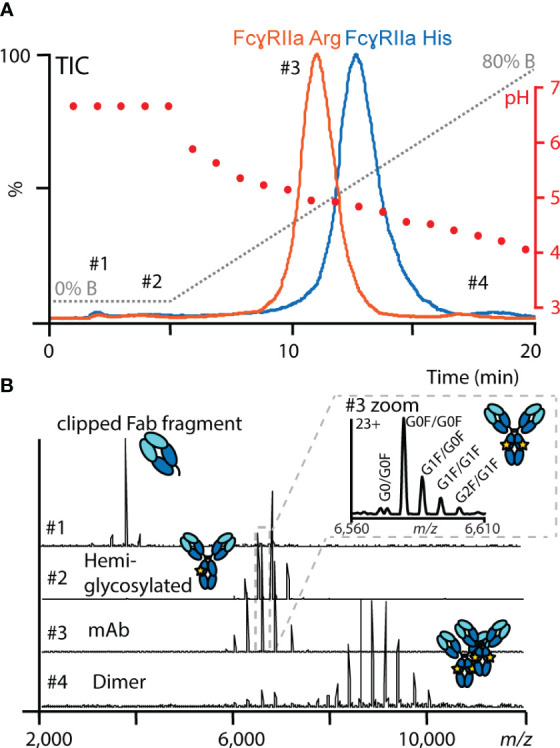
Representative FcγRIIa AC-MS profile of mAb1. **(A)** Total ion chromatograms (TICs) of mAb1 using FcγRIIa His (blue) and FcγRIIa Arg (orange). In addition, the mobile phase (gray) and pH gradient information (red) are displayed. **(B)** Examples of data and spectra quality obtained by online native MS at different retention times.

Sufficient binding and elution of mAb1 under MS-compatible conditions were achieved for both FcγRIIa variants by a simple pH gradient (from pH 6.8 to pH 4) applying a mobile phase system containing 150 mM ammonium acetate and 150 mM acetic acid (**Figure 2A**). The main peak of mAb1 eluted in the linear pH range of the gradient and showed an earlier elution (11 min) on the FcγRIIa Arg column compared to FcγRIIa His (12.5 min). The differences in retention times correlate with the more efficient binding of human IgG1 to FcγRIIa His compared to FcγRIIa Arg (20, 21, 58). Besides the main peak, minor amounts of clipping variants (not retained), hemi-glycosylated variants (elution in isocratic phase) and dimer (increased retention time) were detected and separated in the FcγRIIa AC-MS assays (**Figure 2B**). In addition, the elution order (FcγRIIa Arg < FcγRIIa His) was consistent for all tested CHO cell derived IgG1 mAbs (mAb1 – mAb5), which have the same constant domains (G1m17, Km3) but different variable domains and glycoforms (**Figure S7**), demonstrating the broad applicability of our developed FcγRIIa AC-MS method. Retention time differences in the chromatographic profile between the five mAbs were attributed to the glycosylation profile and the impact of the Fab moiety (**Figure S7**), which are known factors to impact the retention time in Fc receptor affinity chromatography (41). Furthermore, good injection repeatability was achieved for inter- and intraday analysis within a time frame of three months and over 100 injections (stability-studies on-going, **Figure S8**) with the FcγRIIa affinity columns.

Residue 131 of FcγRIIa is located at the CH2 (A) interaction interface (**Figure 1A**). Leu 235 of the lower hinge (CH2 (B)) interacts closely with a hydrophobic binding pocket of FcγRIIa, which is hindered by the His131Arg exchange, and hence, FcγRIIa Arg shows generally a lower binding towards human IgG1 (56). The developed FcγRIIa AC-MS method reflected the expected binding trend. This supports that the applied pH gradient and potential differences in protonation did not impact the general affinity ranking. The involvement of FcγRIIa His/Arg in the binding interface makes it highly relevant to study allotype differences of IgG1 proteoform binding. Most of previous studies on IgG1 interactions only included one of the FcγRIIa allotypes and used different experimental setups (e.g. immobilization or biotinylation), which hampers the comparison of binding studies (10). Previously, only FcγRIIa Arg affinity chromatography using non-MS compatible mobile phases (UV only detection) has been reported (18). In this work, we have introduced novel AC-MS assays for FcγRIIa binding assessment of IgG1, which allow to obtain conclusions on FcγRIIa allotype differences based on the retention time differences. To our knowledge, this is the first report using a FcγRIIa His affinity column. The use of online MS-hyphenation enormously expands the information on proteoforms and product-related impurities obtained from a single experiment. This drastically facilitates data interpretation and enhances the efficiency for comparing antibodies.

### Differential impact of IgG1 Fc glycosylation features on binding to FcγRIIa allotypes resolved by AC-MS

**Figure 3:**
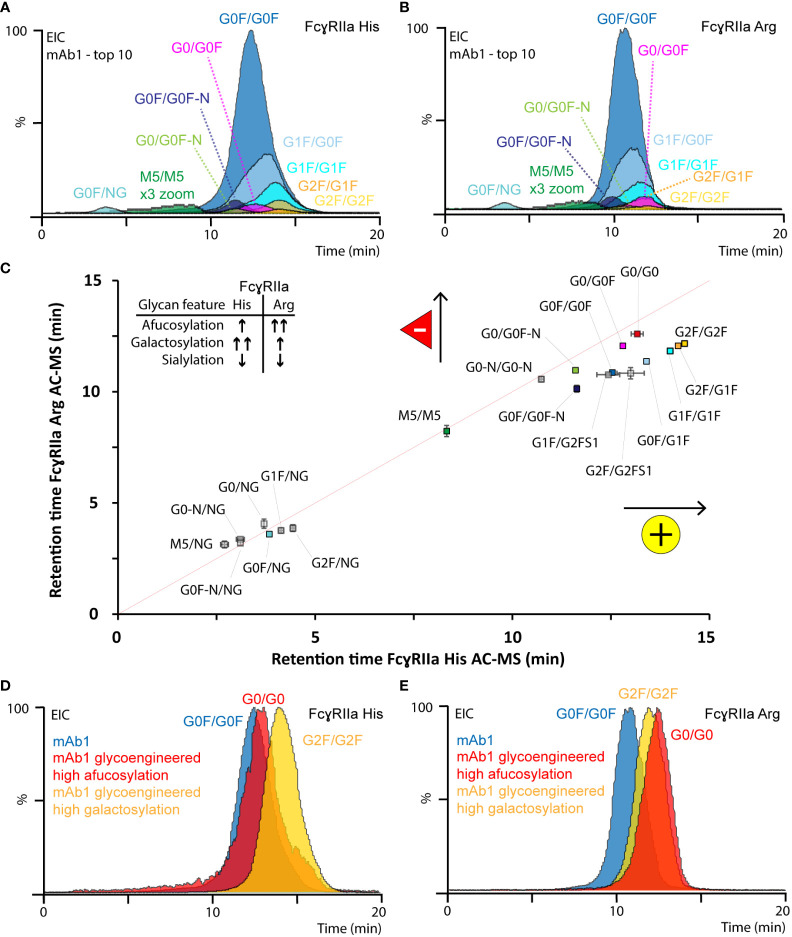
Glycoform-resolved FcγRIIa AC-MS analysis of mAb1. EICs of top ten mAb1 glycoforms (> 1% rel. abundance, **Table S2**) resolved by **(A)** FcγRIIa His and **(B)** FcγRIIa Arg AC-MS. **(C)** Correlation of EIC retention times (error bars represent inter-day standard deviation from n = 3) of all assigned mAb1 glycoforms. The gray line indicates the theoretical retention time, if both FcγRIIa allotypes would have the same affinity. EICs of most-abundant glycoforms from mAb1 and glycoengineered versions of mAb1 analyzed by **(D)** FcγRIIa His and **(E)** FcγRIIa Arg AC-MS.

In total, 20 glycoforms of mAb1 were identified and assessed in detail by AC-MS (**Figure 3**, **Figures S9**, **S10**, **Tables S2**, **S3**). In addition, we used glycoengineered versions of mAb1 and mAb2 to expand and substantiate our findings on glycoform rankings (**Figures S11**-**S13**). The assay showed good reproducibility with respect to retention time stability (average retention time shift below 0.1 min for glycoforms above 1% relative abundance) and relative abundances (average relative standard deviation below 15% for glycoforms above 1% relative abundance) of glycoform EICs (**Figure S10**, **Table S3**). In addition, the high sensitivity and specificity of the MS enabled the study of other minor-abundant (< 1% rel. abundance) glycoforms. The EICs of mAb1 showed overall similar trends for the relative retention time ranking of mAb1 glycoforms with both FcγRIIa allotypes AC (**Figure 3**).

Differences in terminal galactoses (galactosylation) and core fucose (fucosylation) on Fc glycans of mAbs commonly contribute most to the glycosylation microheterogeneity. Glycoforms comprising galactosylation showed an increased retention time (G0F/G0F < G0F/G1F < G1F/G1F < G1F/G2F < G2F/G2F) on both FcγRIIa allotypes. However, the positive effect of galactosylation, relative to afucosylation (the absence of core fucose), was more pronounced for FcγRIIa His AC-MS. The positive impact of galactosylation was independent of the glycosylation macroheterogeneity, fucosylation status and FcγRIIa allotype (**Figure 3**). Interestingly, G1F-derived glycoforms (e.g., G1F/G0F, **Figures 3A, B**) showed a slightly distorted peak shape. We observed a similar behavior of galactosylated glycoforms using FcγRIIIa AC-MS, which was previously attributed to the presence of both α1,3-linked galactose (no effect on binding) and α1,6-linked galactose (increased binding) (59) and could be better resolved by AC when analyzing Fc moieties of mAbs upon upper hinge-cleavage (60). Afucosylation contributed positively to the retention time of both FcγRIIa allotypes (GxF/GxF < Gx/GxF < Gx/Gx), but the effect was stronger for FcγRIIa Arg. For FcγRIIa His, afucosylation only showed a minor positive increase in retention time, which was lower than the impact of galactosylation (**Figure 3**, **Figure S12**, **Table S3**). In contrast, FcγRIIa Arg AC-MS showed that the positive impact of full afucosylation (G0/G0) was even stronger than full galactosylation on doubly fucosylated glycoforms (G2F/G2F) (**Figures 3C, E**, **Figure S12**). The positive impact of galactosylation and afucosylation on mAb1 binding was confirmed by using glycoengineered versions of mAb1 (**Figures 3D, E**, **Figure S11**) and expanded to more glycovariants (glycoengineered versions of mAbs2) with highly homogeneous glycopatterns (**Figure S12**). Previous FcγRIIa binding studies either found no impact of afucosylation at all (17, 20, 21, 23, 27) or a slight positive contribution on the FcγRIIa Arg affinity compared to FcγRIIa His (9, 14, 19, 22, 24). The inconclusive findings in previous studies are likely caused by the inherent heterogeneity of glycosylation and the limited molecular resolution of traditional binding assays. It should be noted that a recent study using glycoform-resolved affinity capillary electrophoresis-MS (ACE-MS) did not find any impact of fucosylation on the mAb - FcγRIIa interaction, independent of the FcγRIIa allotype (27). We performed additional tests using lower ionic strength in our mobile phases (50 mM vs. 150 mM ammonium acetate) to mimic the binding conditions applied in the previous ACE-MS assay (**Figure S12**). However, the glycoform affinity rankings of galactosylation and fucosylation features were consistent for both ionic strengths. Other sources of deviations may come from concentration effects in ACE-MS or the assay setup and should be elaborated in future studies. In contrast, Chung et al. clearly showed the moderate increase (up to 3.6-fold) of afucosylation on FcγRIIa Arg AC-MS binding using highly homogeneous mAb glycoforms and an affinity ranking by enzyme-linked immunoassay, which is in line with our observations (14). Interestingly, another study by Kuhns et al. demonstrated the functional relevance of mAb glycosylation features on FcγRIIa His mediated ADCP using a reporter gene bioassay (15). The authors observed that galactosylation showed a strong positive impact, afucosylation a moderate positive impact and high mannose a strong negative impact, which highlights the functional relevance of our FcγRIIa AC-MS assays.

Other minor abundant glycoforms showed comparable trends for both FcγRIIa allotypes. Agylcosylated mAbs could only be detected for glycoengineered mAb1 (afucosylated) and showed no binding on either of the FcγRIIa variants (**Figure S11**). No binding (aglycosylated) and highly decreased binding (hemi-glycosylated) are in line with previous findings due to highly impaired Fc stability and FcR interactions (27). High mannose glycoforms (i.e., M5/M5) showed increased binding compared to hemi-glycosylated glycoforms and decreased binding compared to complex-type glycoforms (**Figures 3C, D**). The negative impact of high mannose glycoforms on Fc-FcγR interactions, including FcγRIIa, has been demonstrated in several previous studies (16, 22, 26, 27). Glycoforms with mono-antennary structures (-N) showed decreased binding compared to di-antennary structures (**Figure 3C**, **Table S3**). The relative affinity ranking of sialylated glycoforms depended on sialic acid linkage and ionic strength. Low levels (< 0.5%) of singly (α2,3-linked) sialylated glycoforms were detected and assessed in mAb1 (**Figure 3C**, **Table S3**). The sialylated glycoforms (G1F/G2FS1) showed a decreased retention time around G0F/G0F. Glycoengineered variants of mAb2 showed decreased affinity for α2,3-linked sialic acids and increased affinity for α2,6-linked sialic acids for both FcγRIIa allotypes (**Figure S13**). α2,3-linked sialic acids previously showed reduced Fc stability and FcγR binding, whereas α2,6-linked sialic acids increased FcγR binding (44, 61, 62). Of note, the ionic strength of the mobile phases (50 mM vs. 150 mM) had an impact on the relative FcγRIIa Arg affinity ranking of sialylated species (**Figure S12**). Hence, it should be emphasized that the impact of ionic strength needs to be carefully investigated for the relative affinity ranking of sialylated glycoforms.

Asn 297 is located at the FcγRII-IgG1 binding interface and it is known that glycosylation features impact the binding and ADCP (15, 25). The pronounced positive contribution of IgG1 afucosylation on FcγRIIa Arg binding was attributed to the more open state of the CH2 domain, which may contribute to reducing the steric hindrance of Arg131 in the hydrophobic binding pocket (63). Interestingly, the contribution of afucosylation to FcγRIIa Arg binding was higher than galactosylation, which is generally known to stabilize the CH2 domain and FcγR interactions and had a larger contribution to FcγRIIa His binding. In conclusion, FcγRIIa AC-MS provides high molecular resolution, sensitivity, and selectivity, which makes it a powerful tool to unravel the complexity of IgG1 glycoform mixtures and refines the current understanding of IgG1 glycoform affinity rankings.

### Detailed structure-function assessment of Asn 325 deamidated proteoforms on FcγRIIa-mediated ADCP

**Figure 4:**
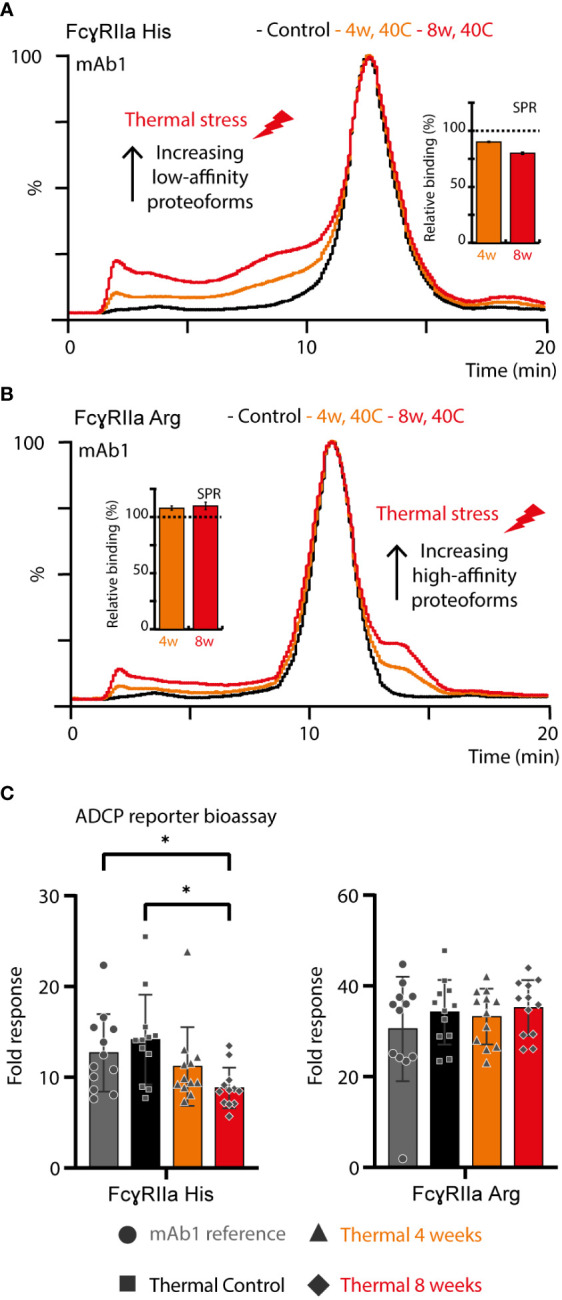
Structure-function assessment of mAb1 upon thermal stress. AC and SPR analysis of mAb1 control, 4 weeks 40°C and 8 weeks 40°C stress sample using **(A)** FcγRIIa His and **(B)** FcγRIIa Arg. Error bars represent standard deviation of technical SPR replicates (n = 2). **(C)** Lymphocyte-based ADCP reporter bioassay of thermally stressed mAb1 on cells exclusively expressing FcγRIIa His or FcγRIIa Arg. Error bars represent the standard deviation of individual technical replicates (n = 12) from two independent ADCP reporter bioassay days. * represent significant differences for p-values < 0.05.

Thermal stress had a noticeable impact on the FcγRIIa AC-MS profile of mAb1 and showed profound differences between the FcγRIIa allotypes (**Figures 4A, B**). Proteoforms induced by thermal stress showed no noticeable shift in the intact mass and eluted, independent of the glycoform, prior to the main peak (lower affinity) in FcγRIIa His AC-MS and with increased retention time (higher affinity) in FcγRIIa Arg AC-MS (**Figures 4**, **S14**). Peptide mapping analysis showed increased level of Asn 325 deamidation, a known thermal stress induced modification (only under mildly acidic conditions) (32, 33), in mAb1 (10.3% at 4 weeks, 18.3% at 8 weeks, compared to 0.7-0.8% in mAb1 Standard and Stress Control) and other PTMs stayed below 5% relative abundance. Orthogonal SPR binding studies confirmed the allotype-specific effect of thermally stressed mAb1 on FcγRIIa binding (FcγRIIa His decreased to 90% ± 0.5% relative binding compared to reference at 4 weeks and 80% ± 1.1% relative binding at 8 weeks, FcγRIIa Arg increased to 108% ± 1.9% relative binding at 4 weeks and 110% ± 3.3% relative binding at 8 weeks, **Figures 4A, B**). In addition, the functional impact of FcγRIIa allotypes-specific binding responses to thermal stress was verified by bioassays applying Jurkat NFAT-luciferase reporter cells expressing exclusively FcγRIIa His or FcγRIIa Arg, which were used as surrogate for FcγRIIa-mediated ADCP (**Figure 4C**). The reporter cells measure the upstream signaling events resulting from FcγRIIa cross-linking, which is the first step of ADCP. FcγRIIa His expressing Jurkat cells showed a significant decrease in activity when mAb1 was exposed to thermal stress for 8 weeks. In contrast, the activity of FcγRIIa Arg expressing Jurkat cells showed no significant difference upon thermal stress of mAb1. Of note, SPR and bioassays measure the combined effects of all mAb1 forms present in the stress samples, including the increased presence of non-potent clipping variants under thermal stress conditions. This may contribute negatively to the overall binding and activity of thermal stressed mAb1 compared to the reference standard and control. Hence, the contribution of Asn 325 deamidated proteoforms to the observed FcγRIIa allotype differences may be slightly underestimated (FcγRIIa Arg) or overestimated (FcγRIIaHis) when analyzing proteoform mixtures. However, the differential FcγRIIa allotype-effect of thermal stress, i.e., Asn 325 deamidation, was in line between AC-MS, SPR and bioassay.

**Figure 5:**
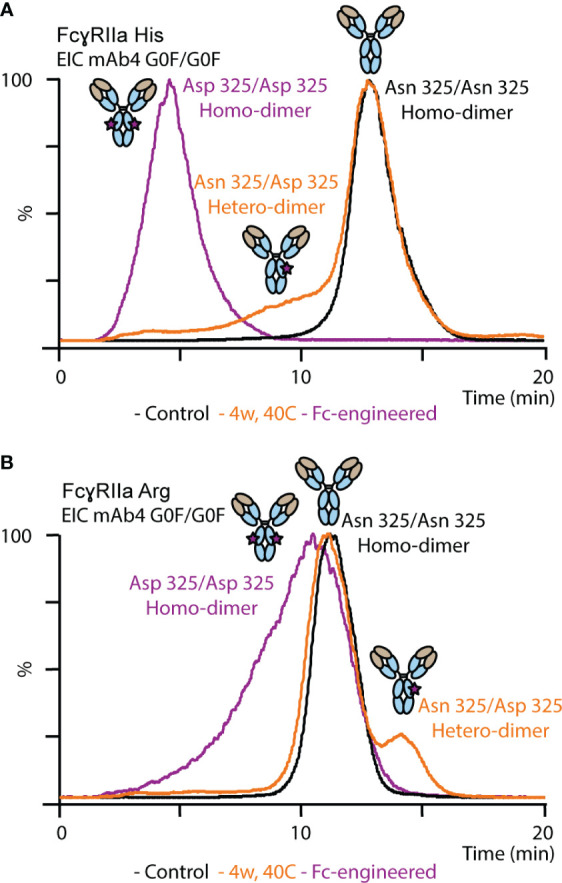
Analysis of mAb4 standard, mAb4 upon thermal stress for 4 weeks and Fc-engineered variant of mAb4 comprising Asp 325 using **(A)** FcγRIIa His and **(B)** FcγRIIa Arg AC-MS.

Proteoforms comprising asymmetric Asn 325 deamidation on only one Fc chain (deamidation hetero-dimer) or symmetrical Asn 325 deamidation on both Fc chains (deamidation homo-dimer) are potentially present upon thermal stress. The majority of the deamidated proteoforms was assigned to deamidation hetero-dimers assuming a random statistical distribution of deamidation (**Figure 5**). In addition, an Fc-engineered mutant of mAb4 was used as control to further support the assignment of the less abundant Asn 325 deamidation homo-dimers (**Figure 5**). The FcγRIIa allotype-dependent difference for Asn 325 deamidation was observed as well for the Fc-engineered Asn 325 deamidation homodimer. The retention time of the homo-dimer was further reduced compared to the hetero-dimer in FcγRIIa His AC-MS. In contrast, the retention time of the homo-dimer was only slightly decreased with a broader peak for FcγRIIa Arg affinity, whereas the hetero-dimer showed increased retention time compared to the unmodified proteoform. Decreased binding of Asn 325 deamidated proteoforms (hetero- and homo-dimers) was described previously for FcγRIIIa interactions (33, 35, 37). Evans et al. demonstrated that asymmetrically modified (deamidation hetero-dimer) was sufficient to drastically reduce the binding and that symmetric degradation (deamidation homo-dimer) further decreased the affinity (35). IgG1 binds FcγRIIa asymmetrically between the CH2 domains and the lower hinge region, similar to FcγRIIIa (**Figure 1A**). Hence, the findings on FcγRIIa His binding differences are in line with previous reports on FcγRIIIa. We obtained novel findings for the impact of proteoform integrity, i.e., increased affinity of the hetero-dimer and slightly reduced affinity for the homo-dimer, on FcγRIIa Arg binding.

**Figure 6:**
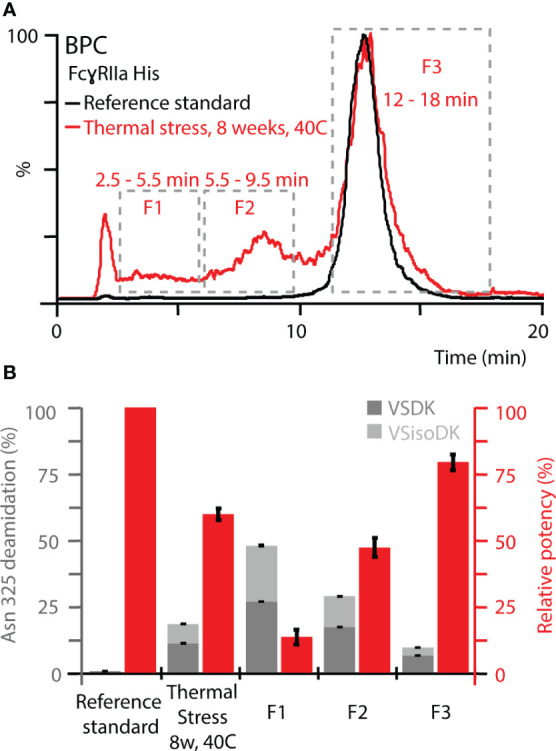
Monocyte-based bioassay and Asn 325 deamidation assessment of affinity fractions from FcγRIIa His. **(A)** Overview of retention time windows for fraction collection. **(B)** Relative potency of fractions (red) and deamidation rates at Asn 325 (gray). Both Asn deamidation products, aspartic acid (VSDK) and iso-aspartic acid (VSisoDK) were assessed based on retention time differences of peptides (**Figure S15**). Error bars represent standard deviation of technical replicates (n = 3). Of note, hemi-glycosylated proteoforms co-eluted with Asn 325 deamidation homo-dimer (F1), which led to a decrease in the overall deamidation rate for the enriched affinity fraction. In addition, the purity of the enriched affinity fractions should be considered with respect to deviations from theoretical assumptions (**Figure S16**).

To further expand the correlation between functional relevance, structural resolution, and retention time in FcγRIIa AC-MS, fractionated thermal stress samples were subjected to bottom-up analysis and a monocyte-derived bioassay. We focused our investigations on the FcγRIIa His column due to its ability to separate deamidation homo- and hetero-dimers, which provides a greater potential as tool to monitor functionally relevant proteoforms, i.e., Asn 325 deamidation variants. An inverse correlation of Asn 325 deamidation and cell-based monocyte activation potency was observed for thermally stressed mAb1 (**Figure 6**). Proteoforms with a higher retention time in FcγRIIa AC-MS exhibited lower levels of Asn 325 deamidation and a higher potency when compared to the reference standard and unstressed thermal control mAb1. In contrast, proteoforms with reduced potency and retention time had higher levels of Asn 325 deamidation. Within the affinity fractions, no preferential enrichment of iso-aspartic acid or aspartic acid deamidation variants was observed, which indicates that both degradation products are equally critical for the FcγRIIa His interaction (**Figures 6**, **S15**). Further, no significant levels of Asn 325 succinimide intermediate was observed, which was attributed to the instability under the applied bottom-up sample preparation conditions (34). In addition, no indications of succinimide forms in AC-MS (intact) were observed under the applied conditions, but future development of AC-MS approaches using Fc moieties (upon IgG1 hinge cleavage) may provide enhanced resolution and specificity to investigate Asn 325 succinimide intermediates (60). We successfully demonstrated functional proteoform separation of our FcγRIIa His AC-MS assay using a monocyte-based ADCP-surrogate bioassay and correlated the results to Asn 325 deamidation products of affinity fractions by bottom-up analysis with increased structural resolution.

### Molecular dynamics simulations reveal allotype- and proteoform-specific hydrogen bond formation for IgG1-FcγRIIa interactions

**Figure 7:**
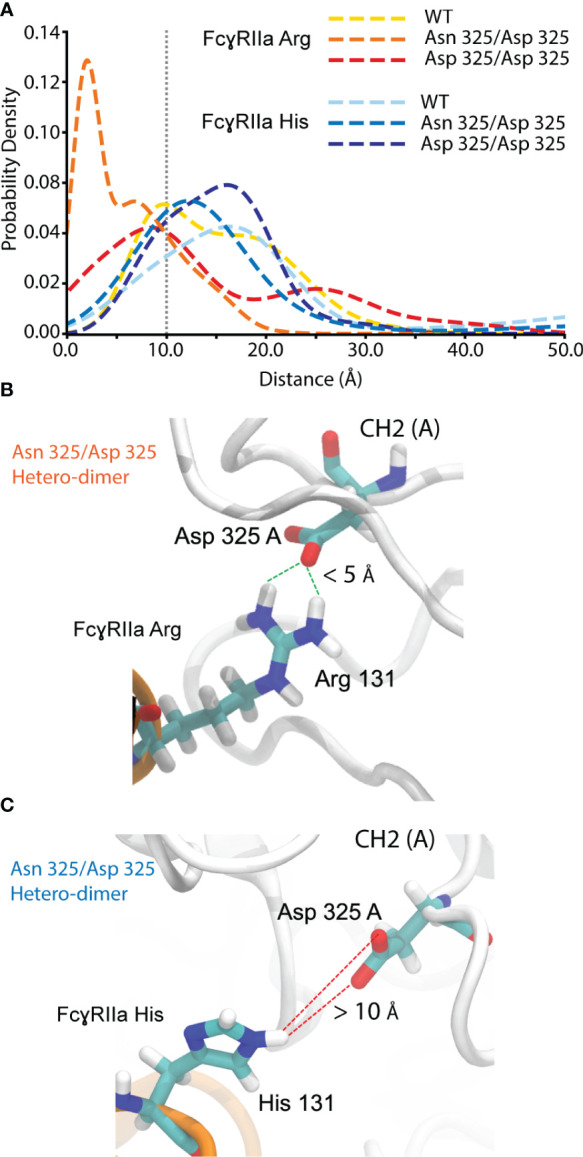
Molecular dynamics simulation for hydrogen bond probability assessment between FcgRIIa residue 131 and IgG1 residue 325. **(A)** Probability density vs. the distance between IgG1 Asn/Asp 325 and FcgRIIa Arg/His 131. Hydrogen bond formation occurs for IgG1 Asn 325 deamidation hetero-dimer and the FcgRIIa Arg allotype. Snapshot visualizing the interaction distance of IgG1 Asp 325 and FcgRIIa **(B)** Arg / **(C)** His 131 for the Asn 325/Asp 325 hetero-dimer.

FcγRIIa interacts asymmetrically with IgG1 at CH2 (A) and CH2 (B) (**Figures 1A**, **S17**). To determine the molecular basis of the observed FcγRIIa allotype-specific differences for deamidated IgG1 proteoform binding trends, we performed MD simulations. This allowed to assess the hydrogen bond formation probabilities of FcγRIIa (Arg/His) interacting with IgG1 as WT, Asn 325 deamidation hetero- or homo-dimers at CH2 (A) (**Figure 7**). A strong hydrogen bond at 1.99 Å was found between FcγRIIa Arg and IgG1 Asp 325, when present as a deamidation hetero-dimer (**Figure 7A**). In contrast, the probability density of a hydrogen bond formation between FcγRIIa Arg and IgG1 Asp 325 was decreased for the IgG1 deamidation homo-dimer, which is in line with our experimental observations by AC-MS (**Figure 5B**). No increased or strong hydrogen bond formation probabilities were observed for the MD simulations of the FcγRIIa His – IgG1 Asp 325 interactions, which supports our findings from FcγRIIa His AC-MS (**Figures 5A**, **7**).

**Figure 8:**
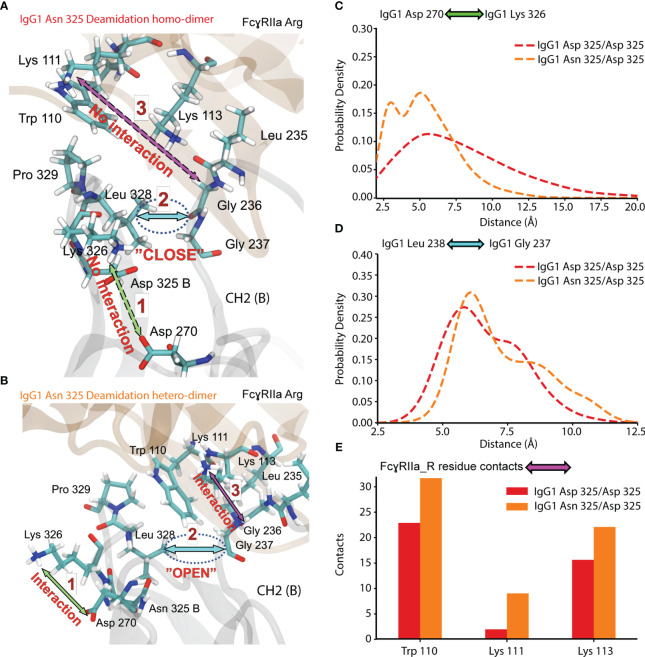
Molecular dynamics of CH2 (B) and FcγRIIa Arg 131 interaction for Asn 325 deamidated proteoforms. **(A)** Residue interaction in IgG1 Asn 325 deamidation homo-dimer. **(B)** Residue interaction in IgG1 Asn 325 deamidation hetero-dimer. Probability density of the **(C)** IgG1 Asp 270 - Lys 326 and **(D)** IgG1 Leu 238 – Gly 237 distance for IgG1 deamidation homo- and hetero-dimer. **(E)** Contacts of FcγRIIa Arg 131 residues to the hydrophobic pocket detected for deamidation homo-dimer (red) and hetero-dimer (orange).

To further investigate the effect of IgG1 proteoform integrity on FcγRIIa Arg binding, we expanded the MD analysis to additional contacts occurring in the Fc (CH2 (B)) of IgG1 (**Figure 8**). The dynamics of a hydrophobic pocket comprising seven residues in the lower hinge and CH2 (Gly 235, Gly 236, Gly 237, Pro 238, Ala 327, Leu 328, Pro 329) were more favorable for the IgG1 Asn 325 deamidation hetero-dimer (**Figure 8**). Formation of the hydrophobic pocket requires the interaction of IgG1 Asp 270 and IgG1 Lys 326 (**Figure 8B**). The initiation of this step is hindered for the IgG1 deamidation homo-dimer due to a competing interaction of IgG1 Asp 325 with IgG1 Lys 326 (**Figures 8A, C**). In contrast, the IgG1 deamidation hetero-dimer comprises one unmodified Fc chain (Asn 325), which enables the first step in the formation of the hydrophobic pocket (**Figures 8B–D**). Trp 110 of FcγRIIa Arg populates the hydrophobic pocket, which further promotes the interaction of FcγRIIa Arg Lys 111 with IgG1 Gly 236. Three FcγRIIa residues (Trp 110, Lys 111 and Lys 113) have more contacts (higher binding) with the hydrophobic pocket for the Asn 325 deamidation hetero-dimer (**Figure 8E**). This is in line with our observation of increased binding of the Asn 325 hetero-dimer in FcγRIIa AC-MS (**Figure 5B**). The fact that the Asn 325 deamidation homo-dimer still showed increased FcγRIIa Arg binding compared to FcγRIIa His (**Figure 5**) was attributed to contributions of the hydrogen bond (IgG1 Asp 325 – FcγRIIa Arg 131, **Figure 7**) and other subtle changes in the interaction. Further, the disturbance in the hydrophobic interactions are the main drivers in reducing the binding of deamidated (hetero- and homo-dimers) IgG1 to FcγRIIa His. In addition, the decreased CH2 stability of the IgG1 Asn 325 deamidation homo-dimer may cause the broader peak observed in FcγRIIa Arg AC-MS (**Figure 5B**). The hydrophobic pocket described here, is one of the three major IgG1-FcγR interaction sites at CH2 (B) (56). A proline sandwich (IgG1 Pro 329, FcγR Trp 87 and Trp 110) is the second major interaction at CH2 (B) (**Figure 1A**), which may contribute to the observed differences as Trp 110 showed different dynamics between deamidated proteoforms (**Figure 8E**). However, the changes in contacts of the Pro 329 were less conclusive compared to the hydrophobic pocket described in **Figure 8**. The third major binding site at CH2 (A), a hydrophobic pocket that generally decreases human IgG1 binding to FcγRIIa Arg, leads to an enhanced affinity of deamidation hetero-dimer due to the Arg 131 – Asp 325 hydrogen bond (**Figure 5**).

### Affinity chromatography-guided approach for functional proteoform assessment

Previous studies of the FcγRIIIa-IgG1 interaction showed the negative effect of Asn 325 deamidation on ADCC (33, 35, 37). FcγRIIIa comprises a His at position 131 and we observed the same negative effect for FcγRIIa His (56). Here, we observed a novel interplay between FcγRIIa allotypes and IgG1 proteoforms. We found that both, the inter-molecular IgG1 Asp 325 hydrogen bond formation probability to FcγRIIa Arg 131 and the intra-molecular formation of the hydrophobic pocket within the Fc of IgG1, are the main drivers for the observed binding selectivity. Our data highlights the relevance of analyzing intact proteoforms rather than bottom-up approaches, to retain the information on deamidation hetero- or homo-dimers. Due to the low mass differences of deamidation (Δ 0.985 Da), intact mass analysis alone will not provide a suitable tool for monitoring deamidation on a proteoform level. Physicochemical separation techniques, such as ion-exchange chromatography, may allow structural separation of thermal stress samples, but suffer from highly increased complexity and low resolution (particularly in stressed samples), which complicates the assignment of functional relevant proteoforms (33). Therefore, novel functional separation techniques such as our new FcγRIIa AC-MS assays are invaluable tools for structure-function studies and have great potential for functional proteoform monitoring. Bioassays have a higher biological relevance compared to cell-free binding assays, since the biological complexity of cellular responses cannot be provided by techniques such as AC-MS (39). Thus, highly sensitive and resolved proteoform readouts as provided by AC-MS should be combined with functional cellular assays to conclude on the biological significance of observed affinity differences. MD simulations were successfully applied to complement the mechanistic differences of our observations.

In our study, we first confirmed the FcγRIIa allotype-specific effect of thermal stress on IgG1 using ADCP reporter bioassays, expressing solely FcγRIIa His or FcγRIIa Arg, which allowed to connect functional proteoform differences solely to the FcγRIIa allotype. In contrast, primary monocytes express FcγRI, FcγRIIb and FcγRIIIa as well (3). The other FcγRs contribute additionally to the effector functions mediated by IgGs. The monocytes used in our study were engineered to express FcγRIIa His but also express FcγRI and potentially low levels of FcγRIIIa. This makes the data more relevant for predicting *in-vivo* activity, but also more complicated to dissect the interplay of IgG1 proteoforms and individual FcγRs. We expect the potential impact from FcγRIIIa-related activity on the overall ADCP to be negligible due to the low abundance compared to FcγRIIa. Interference from FcγRI was excluded since, to our knowledge, no impact of Asn 325 deamidation on FcγRI binding has been reported and we did not observe a difference using SPR (100% relative binding after 4 weeks of thermal stress). In the future, monocytes isolated from patients with different FcγRIIa allotypes (His/His, His/Arg, Arg/Arg) may help to better understand the *in-vivo* relevance of deamidated proteoforms.

The FcγRIIa polymorphism has been associated with different clinical outcomes of diseases (64–66). However, the main underlying mechanisms are currently not well understood. Further, ethnic differences with respect to FcγRIIa polymorphism exist (67, 68). Therefore, it is important to improve the understanding of FcγRIIa-IgG interactions, in particular with respect to PTMs (69). The translation to patients requires more consideration regarding the immune status, i.e., amount and distribution of immune cells, which may overrule the dependence on Fc receptor polymorphism (64). Our findings are fundamental contributions to the functional and structural understanding of IgG1 proteoforms and are highly valuable for defining CQAs of therapeutic mAbs where ADCP contributes to the mechanism of action (70–72).

## Conclusions

In conclusion, we presented an AC-MS guided function-structure approach to streamline the functional and structural assessment of IgG1 proteoforms with respect to FcγRIIa-mediated ADCP activity. Our novel AC-MS assays provide unprecedented selectivity and sensitivity to robustly measure subtle affinity differences of heterogeneous proteoform mixtures. We demonstrated that a single amino acid substitution in the FcγRIIa allotypes differently affected the affinity ranking of IgG1 glycoforms and deamidated IgG1 proteoforms. We showed for the first time an allotype-opposing effect of IgG1 Asn 325 deamidation on FcγRIIa-mediated ADCP and unveiled the underlying structural mechanism. Our study highlights the importance of studying the interplay of PTM combinations, i.e., intact proteoforms, and Fc receptor allotypes, to advance the fundamental immunological understanding of antibody-mediated effector functions. The functional translation of our findings is limited to ADCP-surrogate bioassays and clinical implications remain to be further investigated. Further expansion of the Fc receptor affinity column toolbox and the extension of AC-MS applications to more PTMs, IgG1 allotypes, IgG subclasses and engineered IgGs are expected to drastically enhance the functional understanding of antibody proteoforms. In the future, this will potentially have implications for assessing and monitoring CQAs, engineering next-generation antibodies and providing more individualized patient therapies. We are confident that function-structure approaches guided by AC-MS are invaluable tools for the development of therapeutic antibodies from early-stage research to late-stage extended characterization.

## Data availability statement

The data presented in the study are deposited in the MassIVE repository, accession number MSV000092799.

## Author contributions

SL: Conceptualization, Data curation, Investigation, Methodology, Visualization, Writing – original draft, Writing – review & editing. KM: Conceptualization, Data curation, Investigation, Methodology, Writing – original draft, Writing – review & editing. SL: Conceptualization, Formal Analysis, Methodology, Software, Writing – original draft, Writing – review & editing. KW: Investigation, Methodology, Writing – review & editing. PL: Investigation, Methodology, Writing – review & editing. SP: Methodology, Resources, Writing – review & editing. FK: Methodology, Resources, Writing – review & editing. DR: Resources, Writing – review & editing. LC: Methodology, Writing – review & editing. AK: Conceptualization, Methodology, Resources, Supervision, Writing – review & editing. SI: Conceptualization, Investigation, Methodology, Supervision, Writing – review & editing. AD: Conceptualization, Investigation, Methodology, Supervision, Writing – original draft, Resources, Writing – review & editing. FY: Conceptualization, Supervision, Writing – original draft, Writing – review & editing. TS: Conceptualization, Supervision, Writing – original draft, Writing – review & editing.
