# Supplementary material for: Function-structure approach reveals novel insights on the interplay of Immunoglobulin G 1 proteoforms and Fc gamma receptor IIa allotypes
Source: Front Immunol. 2023 Sep 18;14:1260446. doi: 10.3389/fimmu.2023.1260446 (PMC10544997; doi:10.3389/fimmu.2023.1260446)
Supplement: Supplementary file 1 [file DataSheet_1.docx]

**Supplementary Information**

**Function-structure approach reveals novel insights on the interplay of Immunoglobulin G 1 proteoforms and Fc gamma receptor IIa allotypes**

**Authors:**

**Steffen Lippold^1^, Karishma Mistry^2^, Sunidhi Lenka^3^, Kevin Whang^2^, Peilu Liu^1^, Sebastian Pitschi^4^, Felix Kuhne^4^, Dietmar Reusch^4^, Lance Cadang^1^, Alexander Knaupp^5^, Saeed Izadi^3^, Alexis Dunkle^2^, Feng Yang^1,*^, Tilman Schlothauer^5,*^**

1 Protein Analytical Chemistry, Genentech, A Member of the Roche Group, 1 DNA Way, South San Francisco, California 94080, United States

2 Biological Technologies, Genentech, A Member of the Roche Group, 1 DNA Way, South San Francisco, California 94080, United States

3 Pharmaceutical Development, Genentech, A Member of the Roche Group, 1 DNA Way, South San Francisco, California 94080, United States

4 Pharma Technical Development Europe, Roche Diagnostics GmbH, Penzberg 82377, Germany

5 Pharma Research and Early Development, Roche Innovation Center Munich, Penzberg 82377, Germany

*Corresponding authors e-mail: [yang.feng@gene.com](mailto:yang.feng@gene.com), [tilman.schlothauer@roche.com](mailto:tilman.schlothauer@roche.com)

Contents

[Supplementary Information – Figures 3](#_Toc142576139)

[Figure S1. Sequence of FcγRIIa - Fc fusion protein construct used for FcγRIIa affinity columns. 3](#_Toc142576140)

[Figure S2. Representative HCD MS/MS scan of Asn61 glycopeptide (FcγRIIa His, H4N4F1). 4](#_Toc142576141)

[Figure S3. Representative HCD MS/MS scan of Asn142 glycopeptide (FcγRIIa His, H4N4F1). 4](#_Toc142576142)

[Figure S4. Representative HCD MS/MS scan of Asn61 glycopeptide (FcγRIIa Arg, H3N4F1). 5](#_Toc142576143)

[Figure S5. Representative HCD MS/MS scan of Asn142 glycopeptide (FcγRIIa Arg, H5N4F1S1). 5](#_Toc142576144)

[Figure S6: Representative SPR sensorgrams of FcγRIIa – mAb1 binding analysis 6](#_Toc142576145)

[Figure S7. Generic applicability of FcγRIIa AC-MS method demonstrated for five different IgG1 monoclonal antibodies. 7](#_Toc142576146)

[Figure S8. Overlay of FcγRIIa affinity chromatography profiles for inter- and intra-day analysis of mAb1. 8](#_Toc142576147)

[Figure S9. Representative glycoform assignment visualized on the most abundant charge state of mAb1 using FcγRIIa His AC-MS 9](#_Toc142576148)

[Figure S10. Inter-day analysis (n = 3) of relative abundances of identified glycoforms for mAb1 by FcγRIIa His (blue) and FcγRIIa Arg (green). 10](#_Toc142576149)

[Figure S12. FcγRIIa analysis of glycoengineered mAb2 using TransGLYCIT. 12](#_Toc142576150)

[Figure S13. FcγRIIa His AC-MS analysis of glycoengineered mAb2 with 2,3 or 2,6 sialylation. 13](#_Toc142576151)

[Figure S14. Glycoform-resolved FcγRIIa AC-MS analysis of thermally stressed mAb1. 14](#_Toc142576152)

[Figure S15. Elution time assessment of Asn325 deamidation variants in RP LC-MS/MS. 15](#_Toc142576153)

[Figure S16. Overlay of FcγRIIa His affinity fractions (mAb1) used for monocyte potency assessment (Figure 6). 15](#_Toc142576154)

[Figure S17. Crystal structure of FcγRIIa (in orange) and Fc (in silver) of human IgG1. 16](#_Toc142576155)

[Supplementary Information - Tables 17](#_Toc142576156)

[Table S1. Glycoproteomic analysis of recombinant FcɣRIIa (His and Arg). 17](#_Toc142576157)

[Table S2: Information about the glycan nomenclature used for the main mAb glycoforms used in the main manuscript. 19](#_Toc142576158)

[Table S3. Interday analysis of FcγRIIa AC-MS retention times and relative abundances of resolved glycoforms in mAb1 (n = 3). 20](#_Toc142576159)

# Supplementary Information – Figures

**
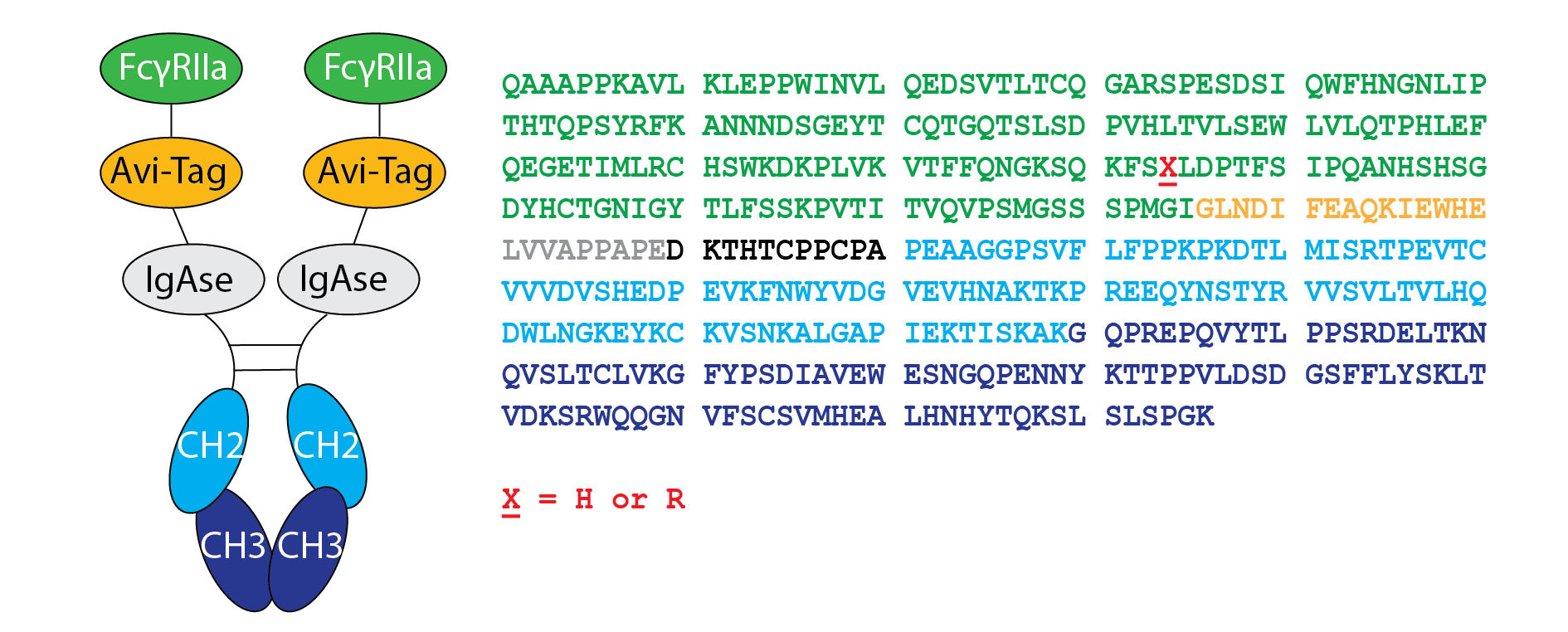
**

## Figure S1. Sequence of FcγRIIa - Fc fusion protein construct used for FcγRIIa affinity columns.

Of note, the numbering for the His / Arg position differs from Figure 1 due to the construct.


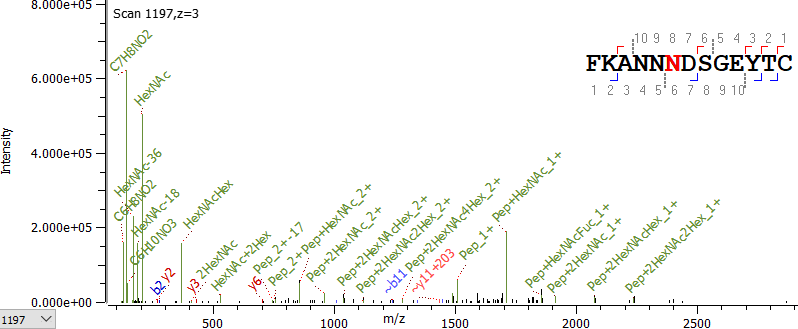


Figure S2. Representative HCD MS/MS scan of Asn61 glycopeptide (FcγRIIa His, H4N4F1). The intact mass of the glycopeptide is 3111.2120 Da, obs. *m/z* 1038.0811 (z = 3), ppm = 3.0. Cysteine is modified by aminoethylation (+43.0422 Da) for tryptic cleavage. For Asn61, a peptide moiety with one missed cleavage site was chosen due to higher intensity.


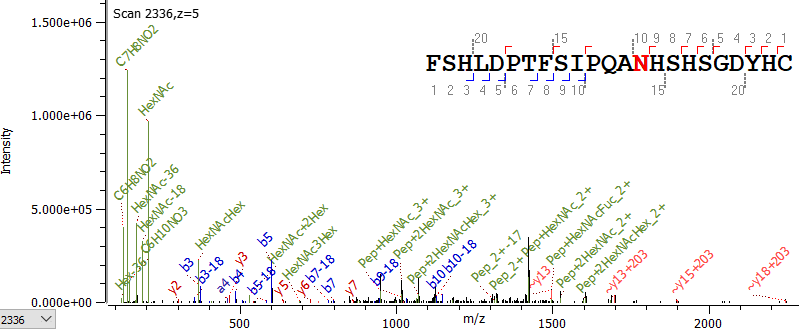


Figure S3. Representative HCD MS/MS scan of Asn142 glycopeptide (FcγRIIa His, H4N4F1). The intact mass of the glycopeptide is 4245.7479 Da, obs. *m/z* 850.1599 (z = 5), ppm = 3.6. Cysteine is modified by aminoethylation (+43.0422 Da) for tryptic cleavage.


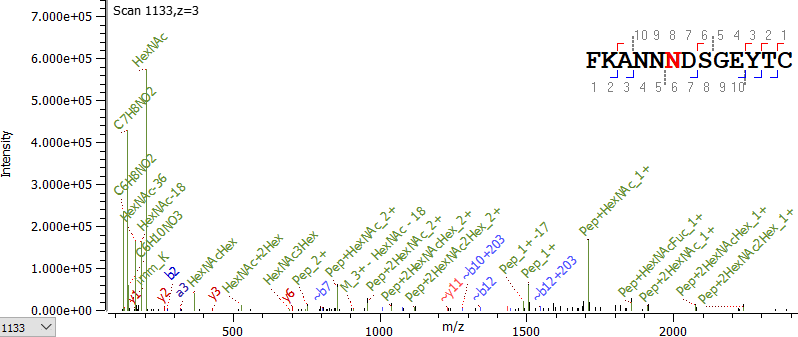


Figure S4. Representative HCD MS/MS scan of Asn61 glycopeptide (FcγRIIa Arg, H3N4F1). The intact mass of the glycopeptide is 2949.1591 Da, obs. *m/z* 984.0629 (z = 3), ppm = 2.6. Cysteine is modified by aminoethylation (+43.0422 Da) for tryptic cleavage. For Asn61, a peptide moiety with one missed cleavage site was chosen due to higher intensity.


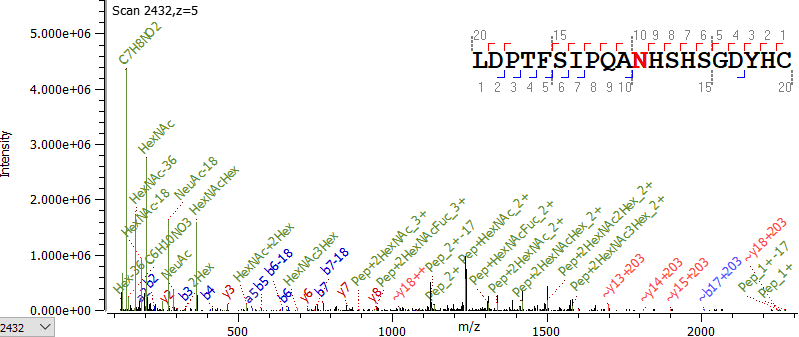


Figure S5. Representative HCD MS/MS scan of Asn142 glycopeptide (FcγRIIa Arg, H5N4F1S1). The intact mass of the glycopeptide is 4327.7367 Da, obs. *m/z* 866.5590 (z = 5), ppm = 5.1. Cysteine is modified by aminoethylation (+43.0422 Da) for tryptic cleavage.


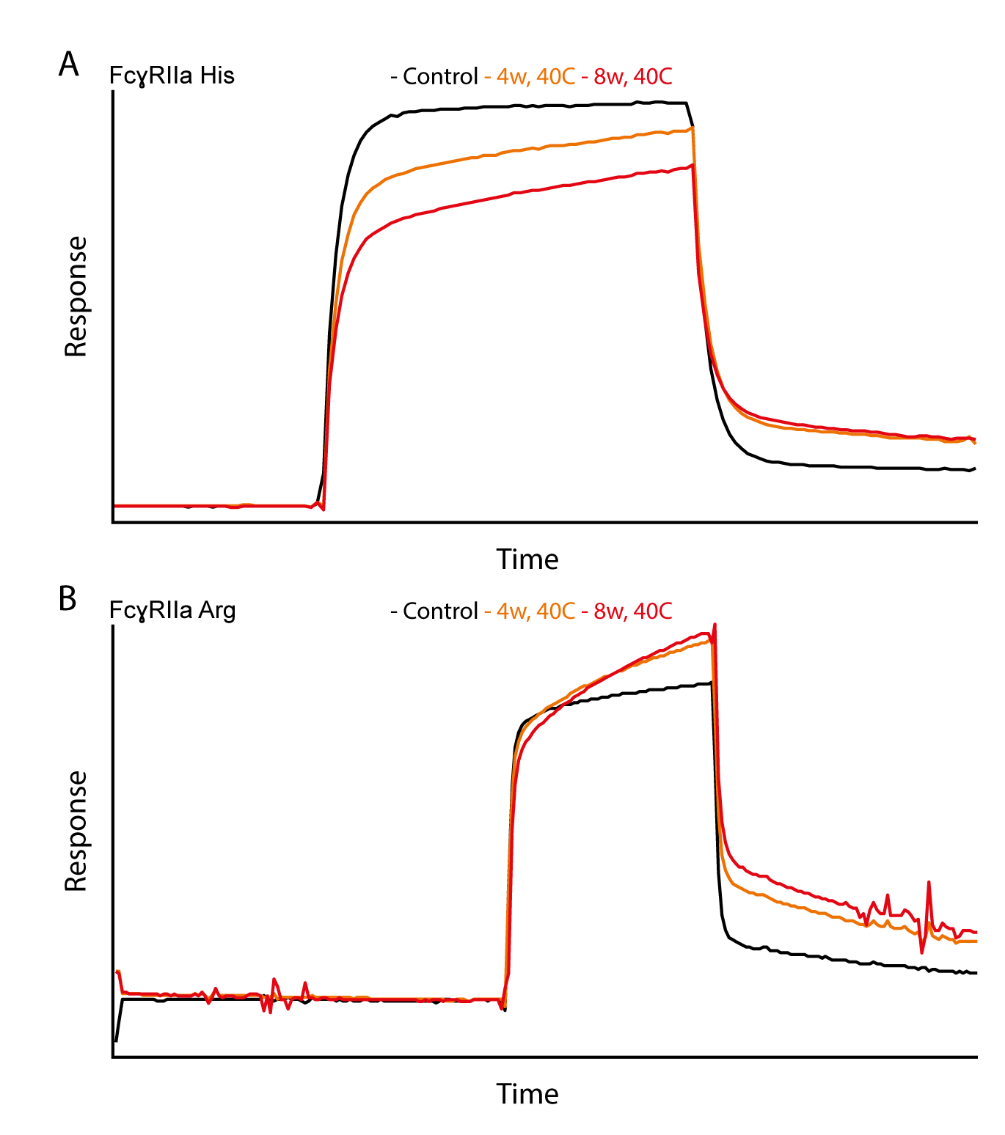


Figure S6: Representative SPR sensorgrams of FcγRIIa – mAb1 binding analysis. A) Binding of thermally stressed mAb1 to FcγRIIa His. B) Binding of thermally stressed mAb1 to FcγRIIa Arg.


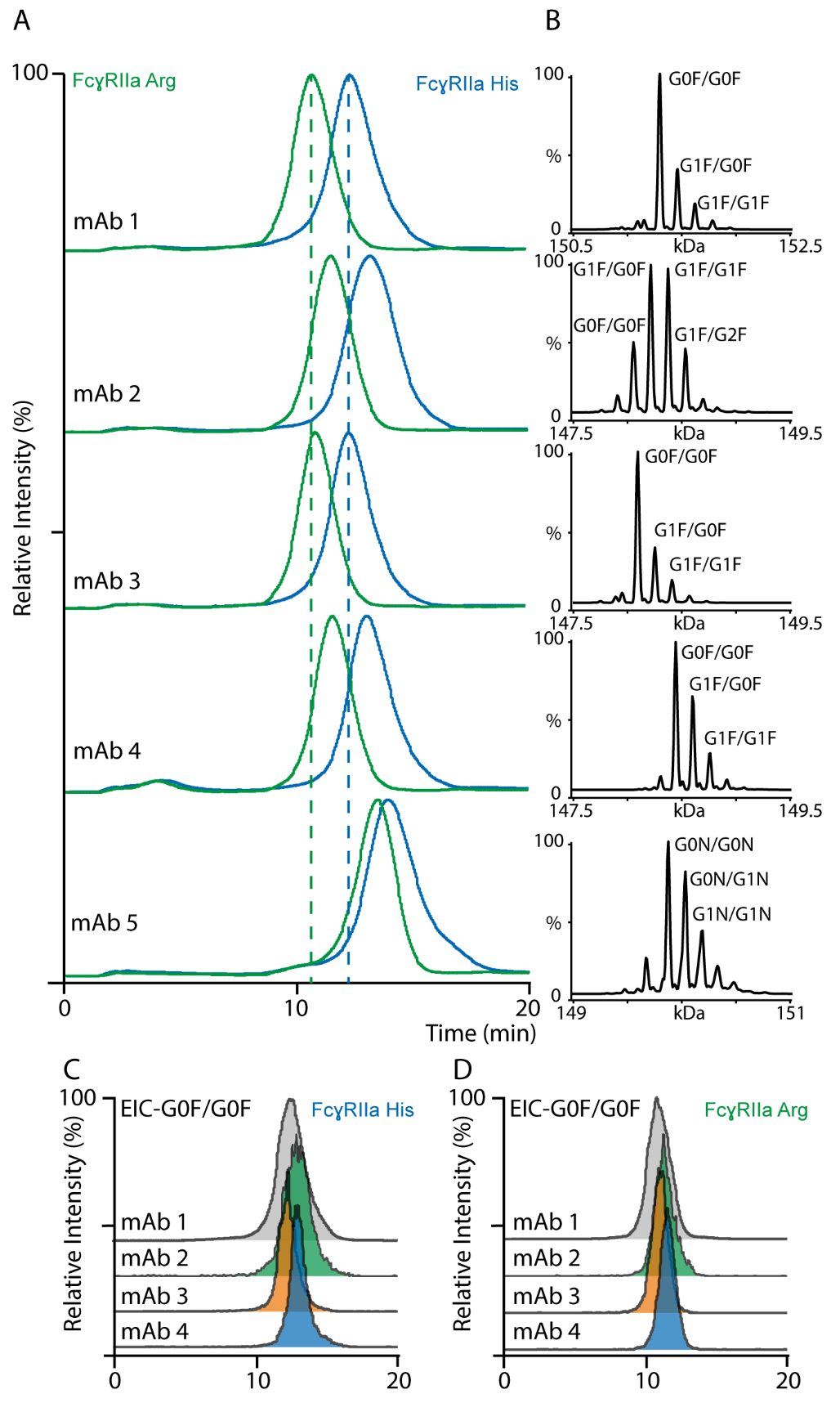


Figure S7. Generic applicability of FcγRIIa AC-MS method demonstrated for five different IgG1 monoclonal antibodies. (A) Representative chromatograms for mAb1 to mAb5 and (B) deconvoluted masses with assigned glycoforms based on representative intact spectra from the main peaks. Extracted ion chromatograms for G0F/G0F from mAb1 to mAb4 demonstrating slight retention time differences based on the Fab portion using (C) FcγRIIa His and (D) FcγRIIa Arg AC-MS.


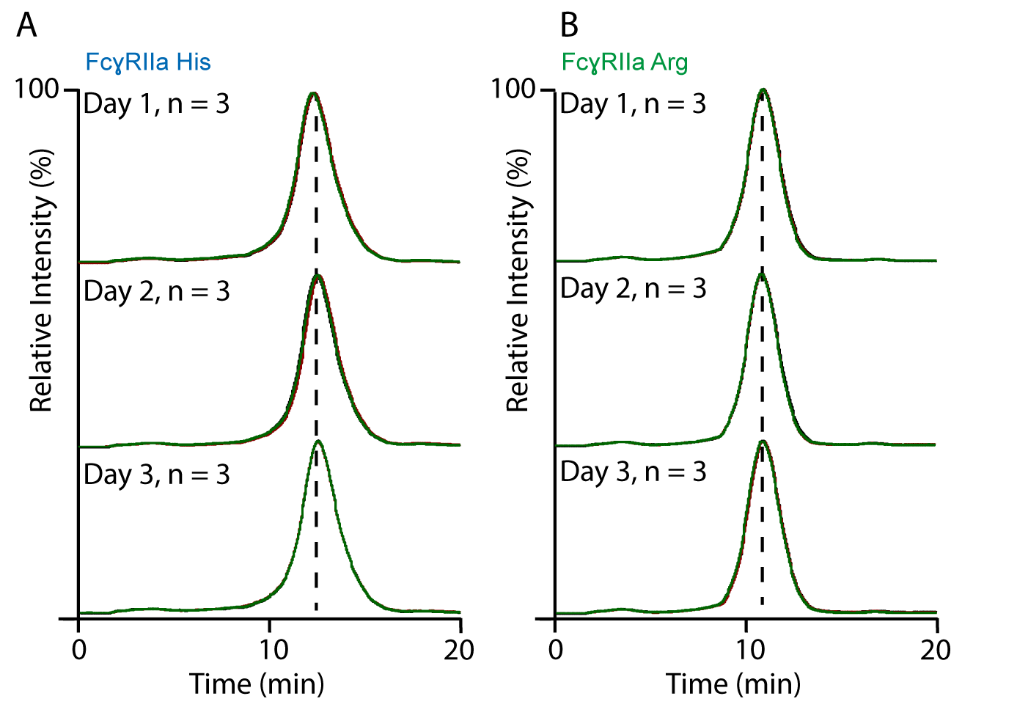


Figure S8. Overlay of FcγRIIa affinity chromatography profiles for inter- and intra-day analysis of mAb1. (A) FcγRIIa His AC overlays and (B) FcγRIIa Arg AC overlays. The three analysis days are spread in a time frame of three months (total n = 9 for each column). The last injection shown in this figure represents injection number 160 (FcγRIIa His) and 85 (FcγRIIa Arg).


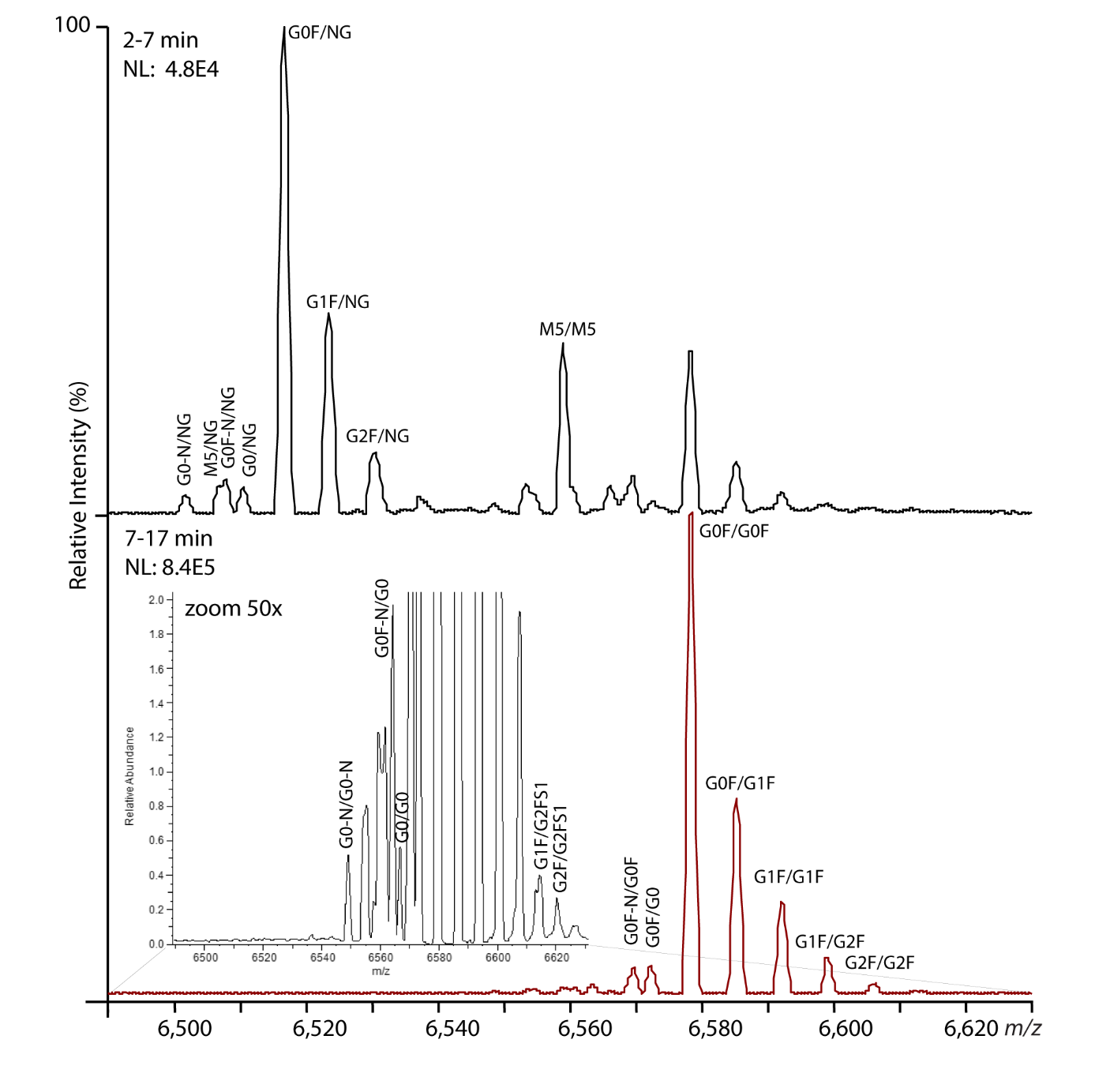


Figure S9. Representative glycoform assignment visualized on the most abundant charge state of mAb1 using FcγRIIa His AC-MS**.** Only well-resolved glycoforms were assigned and used for extracted ion chromatogram analysis and retention time assessment.


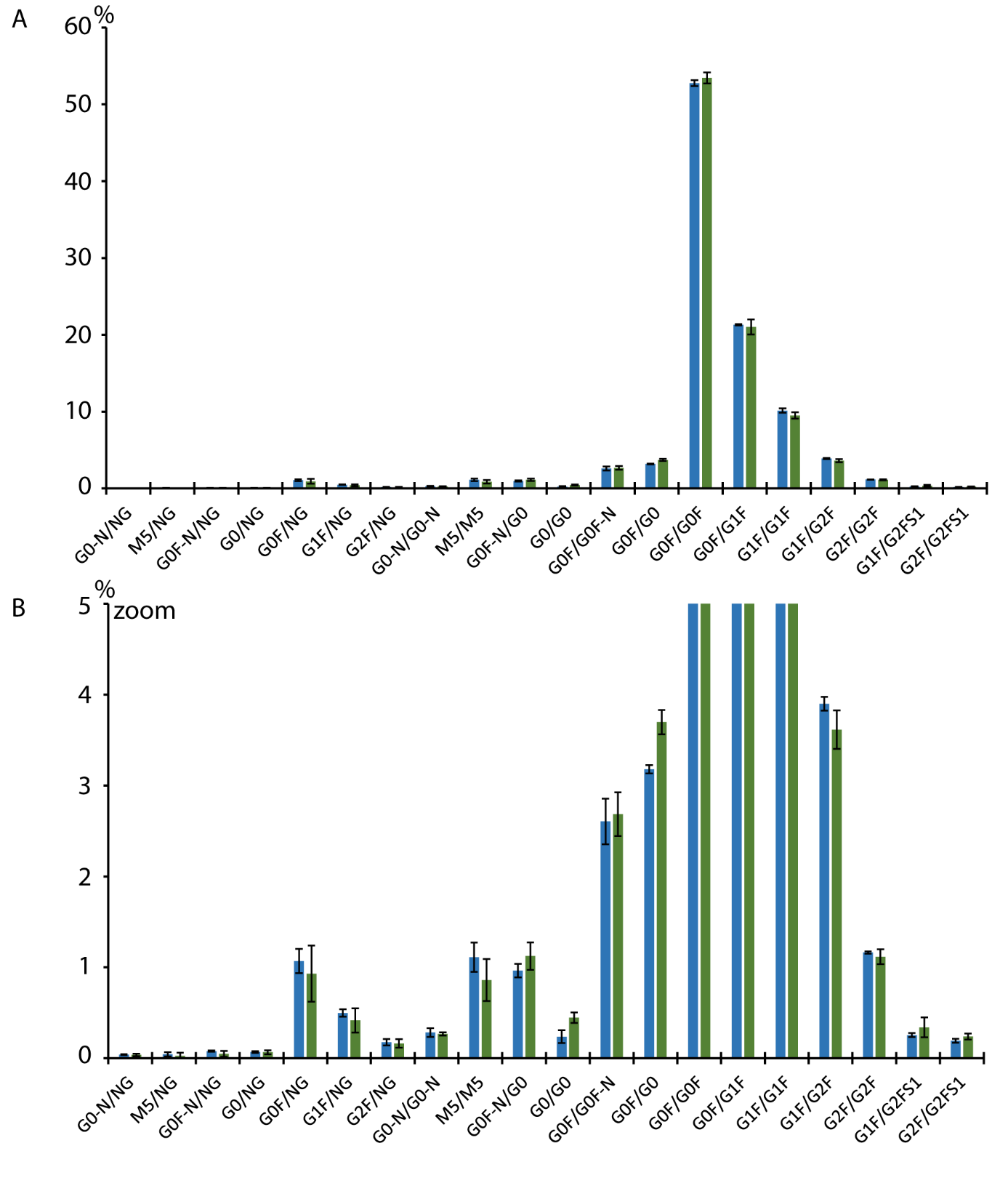


## Figure S10. Inter-day analysis (n = 3) of relative abundances of identified glycoforms for mAb1 by **FcγRIIa** His (blue) and **FcγRIIa** Arg (green).


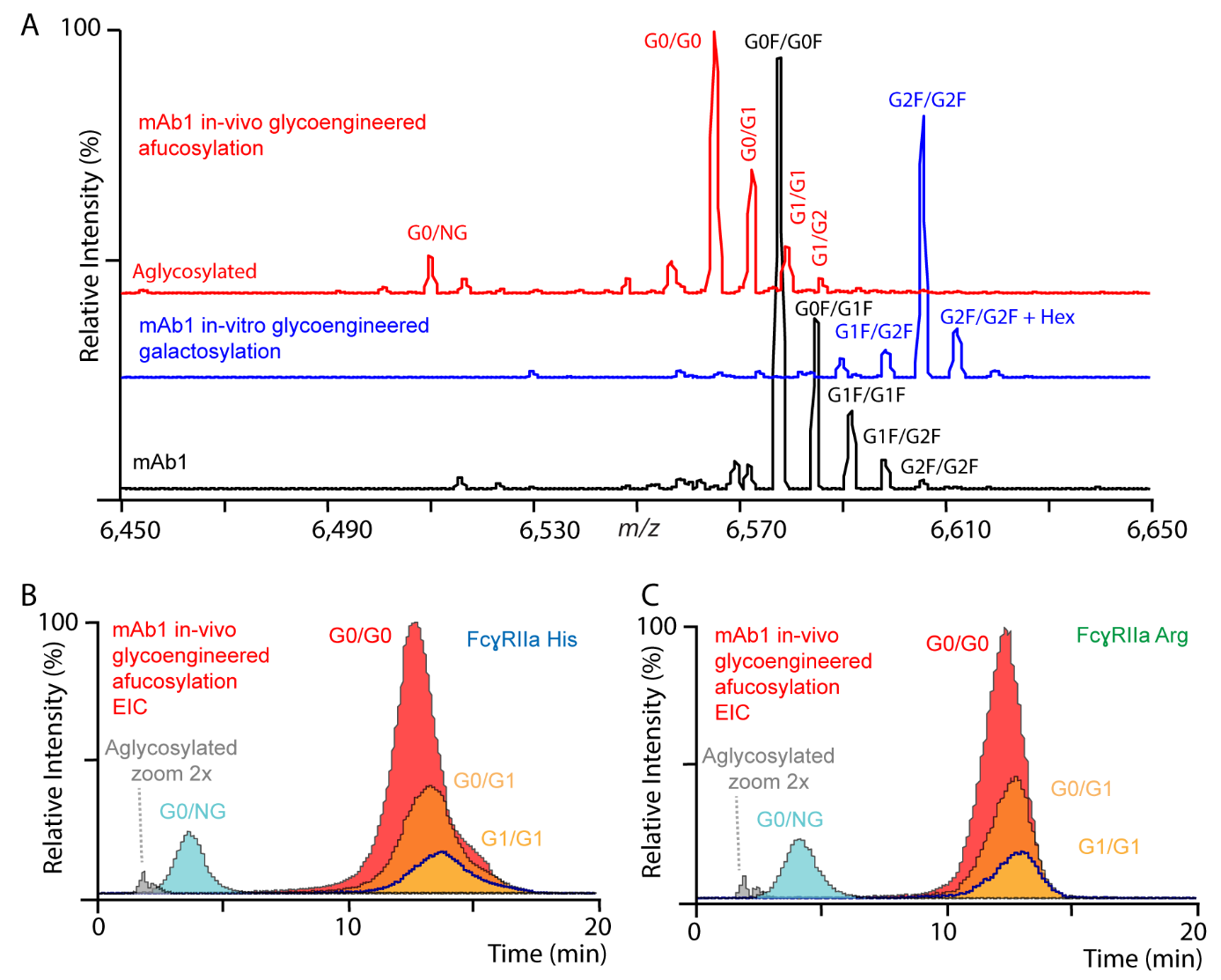
Figure S11. Differences observed for glycoengineered versions of mAb1. (A) Stacked overlay with assignment of relevant glycoforms represented by the most abundant charge state. (B) and (C) show extracted ion chromatograms of selected glycoforms of afucosylated mAb1 on FcγRIIa His AC and FcγRII Arg AC, respectively.


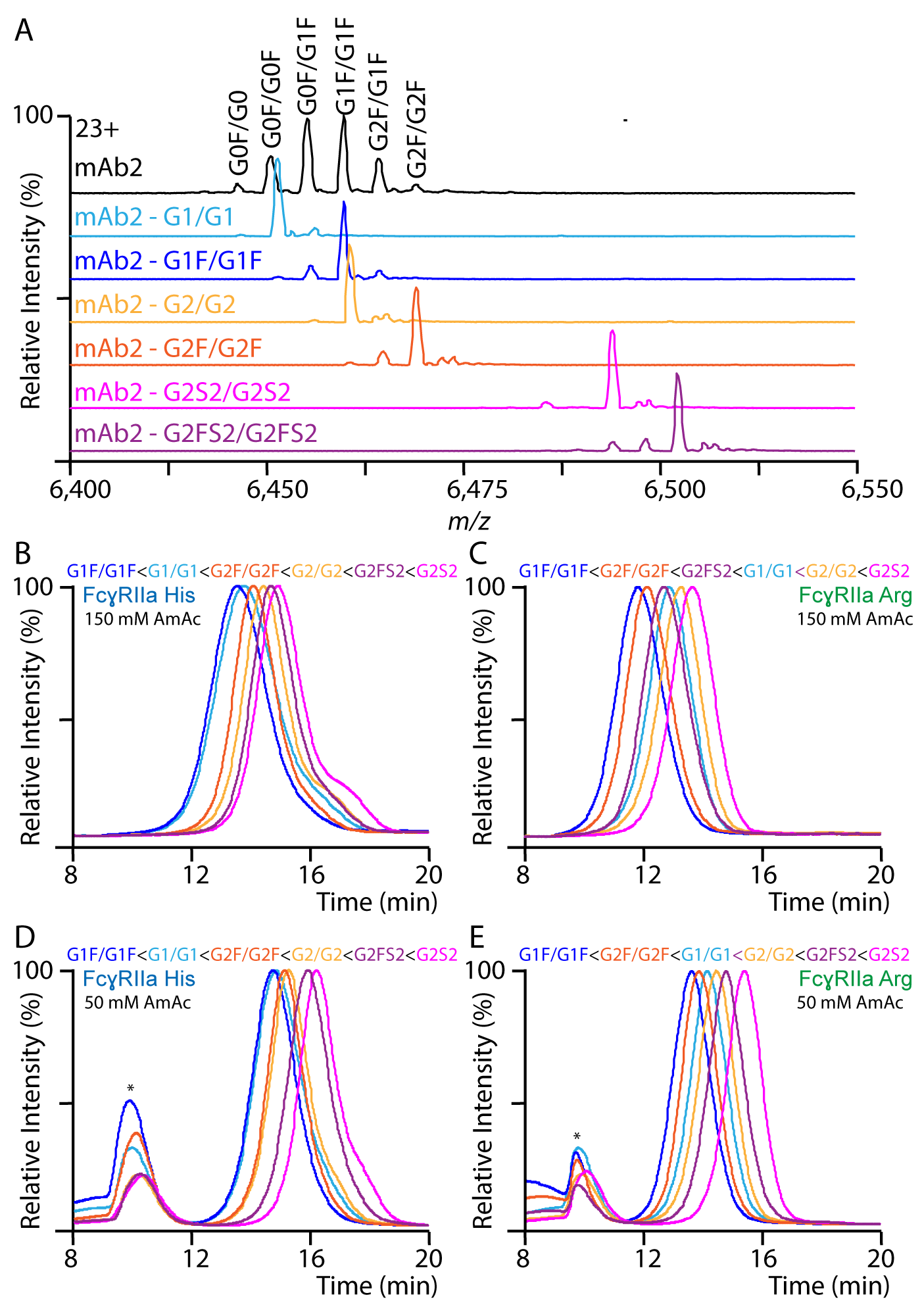


Figure S12. FcγRIIa analysis of glycoengineered mAb2 using TransGLYCIT. (A) Purity of in-vitro glycoengineered versions of mAb2 assessed by the main peak in FcγRIIa. Affinity ranking of glycoengienered mAb2 using 150 mM ammonium acetate for (B) FcγRIIa His and (C) FcγRIIa Arg. Affinity ranking of glycoengienered mAb2 using 50 mM ammonium acetate for (D) FcγRIIa His and (E) FcγRIIa Arg.

* hemi-glycosylated forms including one site with only HexNAc, which is stronger retained with 50 mM ammonium acetate and not not visible in depicted elution window for 150 mM ammonium acetate conditions.


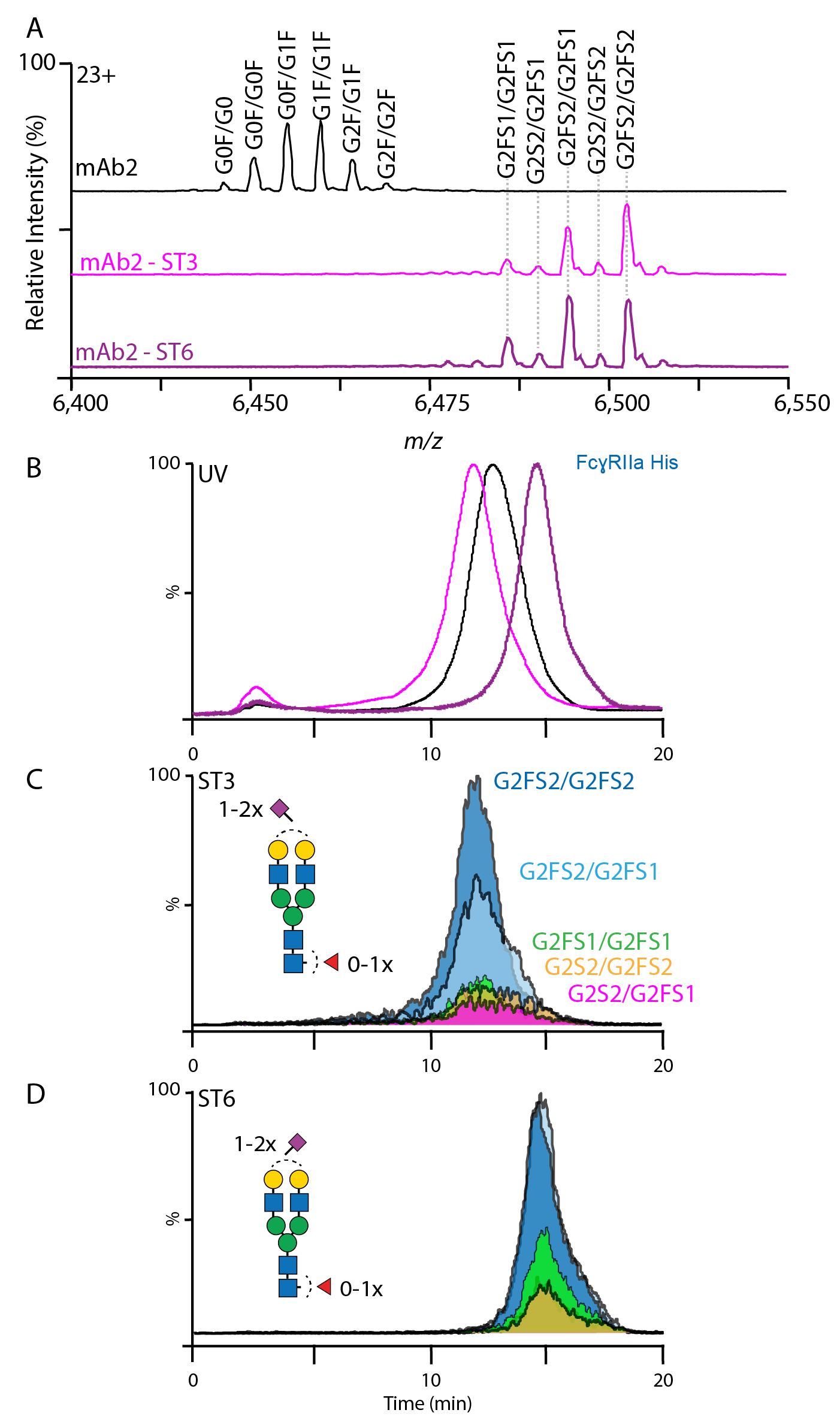


## Figure S13. FcγRIIa His AC-MS analysis of glycoengineered mAb2 with 2,3 or 2,6 sialylation.


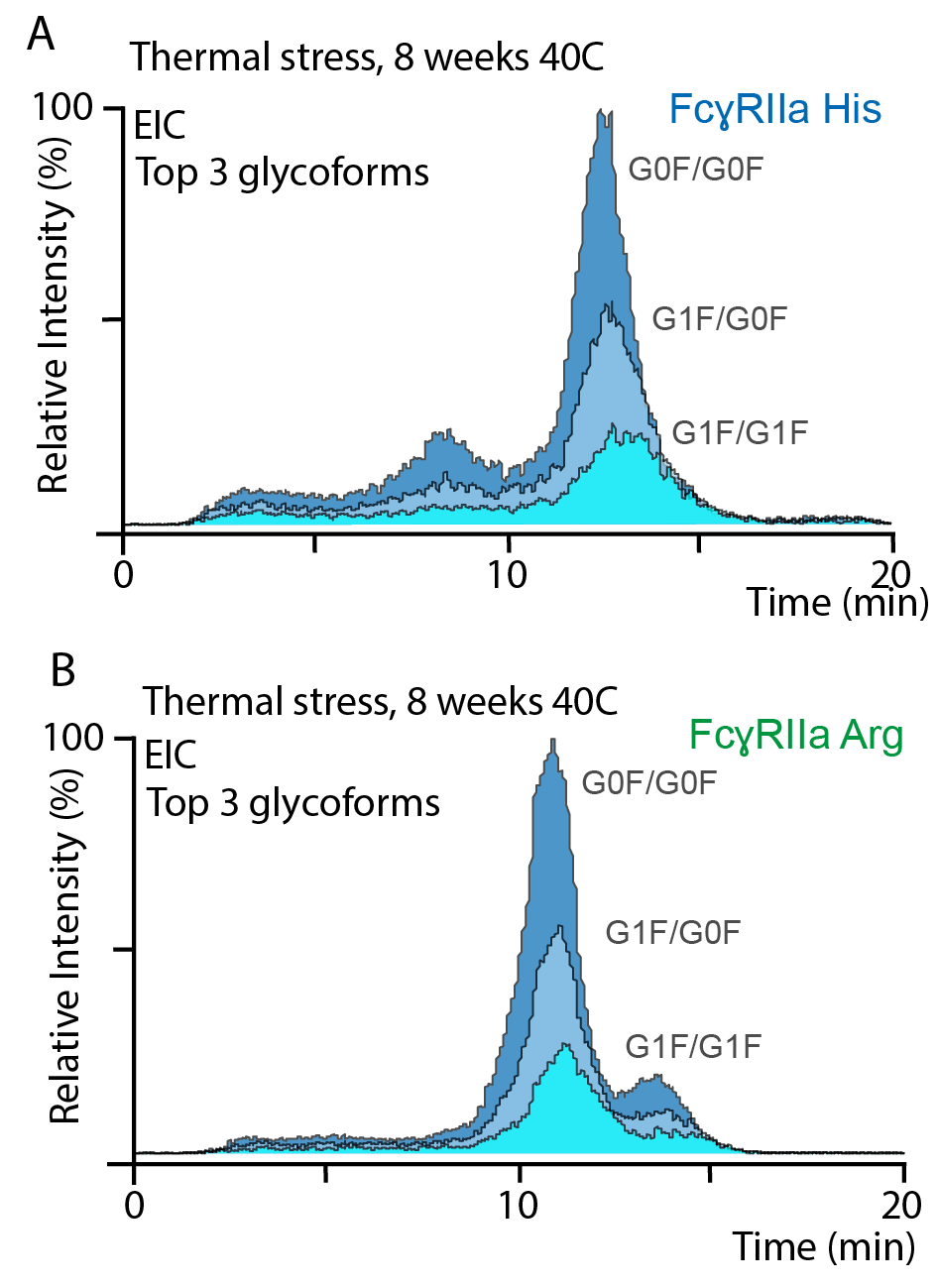


## Figure S14. Glycoform-resolved FcγRIIa AC-MS analysis of thermally stressed mAb1.

Representative EICs of top 3 glycoforms using A) FcγRIIa His and B) FcγRIIa Arg AC-MS.


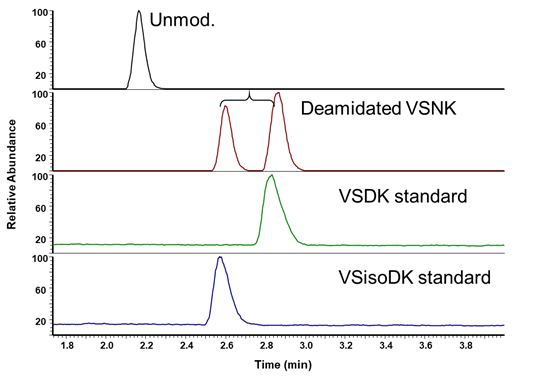


Figure S15. Elution time assessment of tryptic peptides used for the identification of Asn325 deamidation variants analyzed by RP LC-MS/MS. Extracted ion chromatograms of unmodified peptide and deamidated form (top two panels) and total ion chromatograms of VSDK and VSisoDK peptide standards.


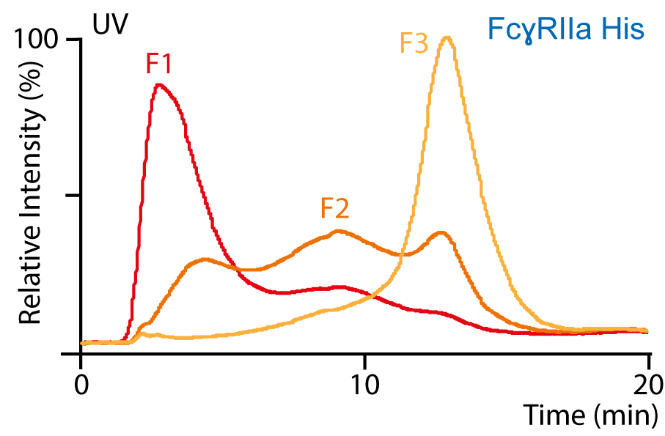


## Figure S16. Overlay of FcγRIIa His affinity fractions (mAb1) used for monocyte potency assessment (Figure 6).

Figure S17. Crystal structure of FcγRIIa (in orange) and Fc (in silver) of human IgG1.

# Supplementary Information - Tables

Table S1. Glycoproteomic analysis of recombinant FcɣRIIa (His and Arg). Assigned glycan compositions as well as relative quantification based on total area normalization for each glycosylation site are listed. Top 10 glycans for each glycosylation site are highlighted in green.

|  | | **Relative Intensity Average (%) \| Standard Deviation (%, n = 3)** | | | | | | | | |
| --- | --- | --- | --- | --- | --- | --- | --- | --- | --- | --- |
|  |  | **His** | | | | | **Arg** | | | |
|  | **Site** | **Asn61** | | **Asn142** | | **Asn61** | | | **Asn142** | |
|  | **Peptide Sequence** | **FKANNNDSGEYTC**** | | **FSHLDPTFSIPQANHS**  **HSGDYHC**** | | **FKANNNDSGEYTC**** | | | **LDPTFSIPQANHS**  **HSGDYHC**** | |
| **Glycan*** | **Monoisotopic Mass (Da)** | **1,504.6253** | | **2,639.1612** | | **1,504.6253** | | | **2,268.0018** | |
| **Not glycosylated** | **-** | 2.9 | 0.56 | - | - | 1.1 | | 0.38 | - | - |
| **H3N2F1** | **1038.3751** | 0.2 | 0.11 | 0.4 | 0.03 | - | | - | - | - |
| **H3N3** | **1095.3966** | 1.0 | 0.05 | 0.9 | 0.19 | 0.2 | | 0.02 | 0.2 | 0.03 |
| **H5N2** | **1216.4228** | 12.0 | 0.74 | 7.9 | 0.86 | 1.8 | | 0.33 | 1.3 | 0.13 |
| **H3N3F1** | **1241.4545** | 2.3 | 1.18 | 3.3 | 0.43 | 0.5 | | 0.16 | 1.0 | 0.11 |
| **H3N4** | **1298.4759** | 1.2 | 0.26 | 0.7 | 0.11 | 0.2 | | 0.00 | 0.1 | 0.01 |
| **H6N2** | **1378.4757** | 0.8 | 0.16 | 0.9 | 0.10 | 0.1 | | 0.02 | 0.2 | 0.03 |
| **H4N3F1** | **1403.5073** | 0.2 | 0.04 | 0.5 | 0.03 | 0.1 | | 0.05 | 0.3 | 0.04 |
| **H3N4F1** | **1444.5339** | 6.2 | 1.82 | 16.4 | 2.03 | 6.0 | | 2.05 | 4.7 | 0.16 |
| **H4N4** | **1460.5288** | 0.3 | 0.01 | 0.2 | 0.02 | 0.1 | | 0.02 | - | - |
| **H3N5** | **1501.5553** | 0.4 | 0.10 | - | - | 0.0 | | 0.01 | - | - |
| **H7N2** | **1540.5285** | 0.9 | 0.04 | 1.0 | 0.12 | 0.1 | | 0.02 | 0.3 | 0.03 |
| **H5N3F1** | **1565.5601** | 0.1 | 0.05 | 0.4 | 0.04 | 0.1 | | 0.02 | 0.2 | 0.02 |
| **H4N4F1** | **1606.5867** | 3.3 | 0.23 | 8.3 | 0.89 | 4.2 | | 1.52 | 3.7 | 0.39 |
| **H3N5F1** | **1647.6132** | 6.1 | 1.26 | 4.0 | 0.19 | 4.9 | | 0.94 | 3.0 | 0.16 |
| **H4N5** | **1663.6081** | 1.3 | 0.25 | - | - | 0.9 | | 0.43 | - | - |
| **H8N2** | **1702.5813** | 0.5 | 0.09 | 0.2 | 0.10 | 0.4 | | 0.03 | 0.1 | 0.00 |
| **H6N3F1** | **1727.6129** | 0.2 | 0.00 | 0.2 | 0.11 | 0.1 | | 0.01 | 0.1 | 0.01 |
| **H4N4F2** | **1752.6446** | 1.0 | 0.10 | 0.5 | 0.05 | 0.8 | | 0.10 | 0.4 | 0.04 |
| **H5N4F1** | **1768.6395** | 3.5 | 0.30 | 4.1 | 0.45 | 6.1 | | 1.64 | 4.2 | 0.46 |
| **H3N5F2** | **1793.6711** | 0.2 | 0.04 | 0.1 | 0.00 | 0.4 | | 0.15 | 0.1 | 0.03 |
| **H4N5F1** | **1809.666** | 5.9 | 1.04 | 2.9 | 0.36 | 8.2 | | 0.63 | 4.0 | 0.48 |
| **H3N6F1** | **1850.6926** | 1.2 | 0.59 | 0.3 | 0.02 | 0.8 | | 0.32 | 0.4 | 0.04 |
| **H4N4F1S1** | **1897.6821** | 3.7 | 0.30 | 4.1 | 2.23 | 1.7 | | 0.43 | 3.1 | 0.62 |
| **H5N4S1** | **1913.677** | - | - | 1.4 | 0.18 | - | | - | 0.4 | 0.12 |
| **H5N4F2** | **1914.6974** | 2.3 | 0.06 | 0.6 | 0.09 | 3.2 | | 0.41 | 1.4 | 0.15 |
| **H6N4F1** | **1930.6923** | 0.1 | 0.00 | - | - | 0.1 | | 0.00 | - | - |
| **H3N5F1S1** | **1938.7086** | 0.5 | 0.11 | - | - | 0.3 | | 0.12 | 0.4 | 0.14 |
| **H4N5F2** | **1955.724** | 1.4 | 0.55 | 0.3 | 0.10 | 3.9 | | 0.94 | 1.2 | 0.08 |
| **H5N5F1** | **1971.7189** | 3.2 | 1.05 | 2.0 | 0.23 | 4.3 | | 1.76 | 3.5 | 0.54 |
| **H3N6F2** | **1996.7505** | 0.1 | 0.00 | - | - | 0.8 | | 0.36 | 0.1 | 0.01 |
| **H4N6F1** | **2012.7454** | 0.6 | 0.04 | - | - | 0.6 | | 0.17 | 0.4 | 0.05 |
| **H5N4F1S1** | **2059.7349** | 15.7 | 0.34 | 23.1 | 1.32 | 15.9 | | 2.21 | 27.6 | 2.18 |
| **H6N4S1** | **2075.7298** | - | - | 0.1 | 0.07 | - | | - | 0.2 | 0.02 |
| **H4N5F1S1** | **2100.7614** | 3.8 | 0.48 | 1.2 | 0.14 | 5.8 | | 1.21 | 4.0 | 0.16 |
| **H5N5F2** | **2117.7768** | 0.4 | 0.11 | 0.3 | 0.03 | 1.1 | | 0.42 | 0.8 | 0.15 |

**Continuation Table S1:**

|  | | **Relative Intensity Average (%) \| Standard Deviation (%, n = 3)** | | | | | | | |
| --- | --- | --- | --- | --- | --- | --- | --- | --- | --- |
|  |  | **His** | | | | **Arg** | | | |
|  | **Site** | **Asn61** | | **Asn142** | | **Asn61** | | **Asn142** | |
|  | **Peptide Sequence** | **FKANNNDSGEYTC**** | | **FSHLDPTFSIPQANHS**  **HSGDYHC**** | | **FKANNNDSGEYTC**** | | **LDPTFSIPQANHS**  **HSGDYHC**** | |
| **Glycan*** | **Monoisotopic Mass (Da)** | **1,504.6253** | | **2,639.1612** | | **1,504.6253** | | **2,268.0018** | |
| **H6N5F1** | **2133.7717** | 2.7 | 0.36 | - | - | 1.8 | 0.11 | 0.9 | 0.03 |
| **H5N6F1** | **2174.7982** | 0.8 | 0.13 | - | - | 1.5 | 0.15 | 0.4 | 0.05 |
| **H4N4F1S2** | **2188.7775** | - | - | - | - | 0.1 | 0.13 | - | - |
| **H5N4S2** | **2204.7724** | - | - | 0.5 | 0.04 | 2.1 | 1.53 | 0.7 | 0.04 |
| **H5N4F2S1** | **2205.7928** | 1.7 | 0.14 | 1.3 | 0.15 | 2.4 | 0.46 | 2.1 | 0.04 |
| **H4N7F1** | **2215.8248** | 0.1 | 0.01 | - | - | 0.4 | 0.09 | - | - |
| **H4N5F2S1** | **2246.8194** | 1.1 | 0.02 | 0.2 | 0.02 | 2.0 | 0.49 | 0.6 | 0.05 |
| **H5N5F1S1** | **2262.8143** | 1.3 | 0.11 | 1.8 | 0.04 | 3.2 | 0.48 | 4.9 | 0.38 |
| **H6N5F2** | **2279.8296** | 0.5 | 0.14 | - | - | 0.6 | 0.09 | 0.2 | 0.03 |
| **H5N6F2** | **2320.8561** | 0.3 | 0.06 | - | - | 0.5 | 0.09 | - | - |
| **H6N6F1** | **2336.8511** | - | - | - | - | 0.2 | 0.07 | 0.4 | 0.03 |
| **H5N4F1S2** | **2350.8303** | 3.3 | 0.04 | 5.8 | 0.46 | 3.9 | 0.10 | 6.6 | 0.16 |
| **H4N5F1S2** | **2391.8568** | 0.3 | 0.02 | 0.3 | 0.03 | 1.0 | 0.11 | 1.0 | 0.05 |
| **H5N5F2S1** | **2408.8722** | - | - | - | - | 0.2 | 0.04 | 0.3 | 0.01 |
| **H6N5F1S1** | **2424.8671** | 1.6 | 0.43 | 1.7 | 0.69 | 1.3 | 0.40 | 3.9 | 0.21 |
| **H5N6F1S1** | **2465.8936** | 0.6 | 0.35 | - | - | 0.9 | 0.06 | 0.4 | 0.09 |
| **H6N6F2** | **2482.909** | - | - | 0.2 | 0.02 | 0.2 | 0.02 | 0.6 | 0.05 |
| **H5N5F1S2** | **2553.9097** | - | - | 0.3 | 0.14 | - | - | 0.8 | 0.06 |
| **H6N5F2S1** | **2570.925** | 0.3 | 0.03 | 0.3 | 0.15 | - | - | 0.1 | 0.01 |
| **H5N6F2S1** | **2611.9515** | - | - | - | - | 0.3 | 0.06 | 0.6 | 0.09 |
| **H6N6F1S1** | **2627.9465** | - | - | - | - | 1.6 | 0.34 | 3.2 | 0.40 |
| **H6N5F1S2** | **2715.9625** | 1.2 | 0.06 | - | - | 0.7 | 0.14 | 0.3 | 0.04 |
| **H5N6F1S2** | **2756.989** | 0.2 | 0.03 | - | - | - | - | 0.4 | 0.03 |
| **H7N6F1S1** | **2789.9993** | - | - | - | - | - | - | 0.3 | 0.03 |
| **H6N5F2S2** | **2862.0204** | - | - | - | - | - | - | 0.9 | 0.13 |
| **H6N6F1S2** | **2919.0419** | - | - | - | - | - | - | 0.1 | 0.01 |
| **H7N6F2S1** | **2936.0572** | - | - | 1.0 | 0.10 | - | - | 1.5 | 0.07 |
| **H6N5F1S3** | **3007.0579** | - | - | 0.2 | 0.05 | 0.2 | 0.03 | 0.7 | 0.01 |
| **H7N6F1S2** | **3081.0947** | 0.2 | 0.01 | - | - | - | - | 0.2 | 0.02 |
| **H6N6F1S3** | **3210.1373** | - | - | - | - | - | - | 0.1 | 0.01 |
| **H7N6F1S3** | **3,372.1902** | - | - | 0.3 | 0.05 | - | - | 0.4 | 0.07 |

***** H = hexose, N = *N*-acetylhexosamine, F = deoxyhexose, S = *N*-acetylneuraminic acid

** Cysteines modified by aminoethylation (+ 43.0422 Da)

## Table S2: Information about the glycan nomenclature used for the main mAb glycoforms used in the main manuscript.

| **Nomenclature*** | **Composition**** | **Average mass (Da)** | **Structure***** |
| --- | --- | --- | --- |
| NG | - | 0 | Not glycosylated |
| G0-N | H3N3 | 1096.0 | **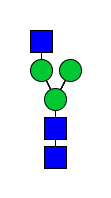** |
| M5 | H5N2 | 1217.1 | **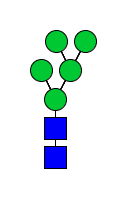** |
| G0F-N | H3N3F1 | 1242.1 | **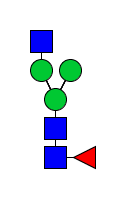** |
| G0 | H3N4 | 1299.2 | **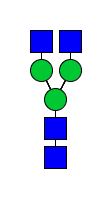** |
| G0F | H3N4F1 | 1445.3 | **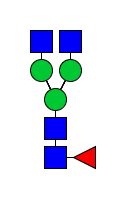** |
| G1 (1,3 or 1,6) | H4N4 | 1461.3 | **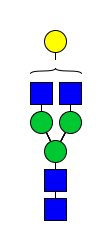** |
| G1F (1,3 or 1,6) | H4N4F1 | 1607.5 | **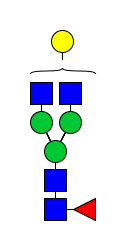** |
| G2 | H5N4 | 1623.5 | **** |
| G2F | H5N4F1 | 1769.6 | **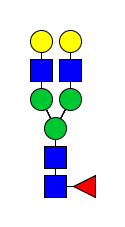** |
| G2FS1 | H5N4F1S1 | 2060.9 | **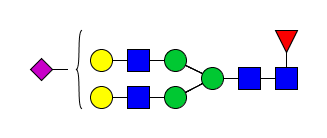** |

***** NG = non-glycoslated, M = mannose, G = galactose, F = core fucose

****** H = hexose, N = *N*-acetylhexosamine, F = deoxyhexose, S = *N*-acetylneuraminic acid

******* Structures based on existing knowledge of Fc glycosylation in CHO cell produced IgG1 mAbs

## Table S3. Interday analysis of FcγRIIa AC-MS retention times and relative abundances of resolved glycoforms in mAb1 (n = 3).

|  | His |  |  |  | Arg |  |  |  |
| --- | --- | --- | --- | --- | --- | --- | --- | --- |
| Glycoform | RT (min) | RT STDEV (min) | Rel. % | Rel. % STDEV | RT (min) | RT STDEV (min) | Rel. % | Rel. % STDEV |
| G0-N/NG | 3.1 | 0.10 | 0.04 | 0.005 | 3.4 | 0.06 | 0.04 | 0.014 |
| M5/NG | 2.7 | 0.10 | 0.04 | 0.024 | 3.1 | 0.15 | 0.03 | 0.033 |
| G0F-N/NG | 3.1 | 0.00 | 0.08 | 0.007 | 3.2 | 0.10 | 0.05 | 0.032 |
| G0/NG | 3.7 | 0.00 | 0.07 | 0.009 | 4.1 | 0.21 | 0.07 | 0.022 |
| G0F/NG | 3.8 | 0.06 | 1.07 | 0.134 | 3.6 | 0.10 | 0.93 | 0.309 |
| G1F/NG | 4.1 | 0.06 | 0.50 | 0.041 | 3.8 | 0.12 | 0.42 | 0.134 |
| G2F/NG | 4.4 | 0.06 | 0.17 | 0.035 | 3.9 | 0.15 | 0.16 | 0.047 |
| G0-N/G0-N | 10.7 | 0.06 | 0.28 | 0.049 | 10.6 | 0.12 | 0.27 | 0.018 |
| M5/M5 | 8.3 | 0.06 | 1.11 | 0.161 | 8.2 | 0.25 | 0.86 | 0.232 |
| G0F-N/G0 | 11.6 | 0.00 | 0.96 | 0.074 | 11.0 | 0.06 | 1.12 | 0.152 |
| G0/G0 | 13.2 | 0.15 | 0.24 | 0.071 | 12.6 | 0.10 | 0.45 | 0.057 |
| G0F/G0F-N | 11.6 | 0.06 | 2.60 | 0.251 | 10.1 | 0.15 | 2.68 | 0.240 |
| G0F/G0 | 12.8 | 0.00 | 3.18 | 0.046 | 12.1 | 0.06 | 3.70 | 0.134 |
| G0F/G0F | 12.5 | 0.06 | 52.75 | 0.390 | 10.9 | 0.06 | 53.44 | 0.718 |
| G0F/G1F | 13.4 | 0.00 | 21.31 | 0.092 | 11.4 | 0.06 | 21.03 | 0.984 |
| G1F/G1F | 14.0 | 0.00 | 10.13 | 0.295 | 11.8 | 0.06 | 9.50 | 0.420 |
| G1F/G2F | 14.2 | 0.00 | 3.90 | 0.075 | 12.1 | 0.06 | 3.62 | 0.212 |
| G2F/G2F | 14.4 | 0.06 | 1.16 | 0.012 | 12.2 | 0.06 | 1.12 | 0.081 |
| G1F/G2FS1 | 12.4 | 0.29 | 0.25 | 0.022 | 10.8 | 0.06 | 0.34 | 0.109 |
| G2F/G2FS1 | 13.0 | 0.35 | 0.19 | 0.021 | 10.8 | 0.25 | 0.24 | 0.032 |
